# The Role of Exercise, Diet, and Cytokines in Preventing Obesity and Improving Adipose Tissue

**DOI:** 10.3390/nu13051459

**Published:** 2021-04-25

**Authors:** Muhammed Mustafa Atakan, Şükran Nazan Koşar, Yasemin Güzel, Hiu Tung Tin, Xu Yan

**Affiliations:** 1 Division of Exercise Nutrition and Metabolism, Faculty of Sport Sciences, Hacettepe University, 06800 Ankara, Turkey; muhammed.atakan@hacettepe.edu.tr (M.M.A.); nazank@hacettepe.edu.tr (Ş.N.K.); yasmin@hacettepe.edu.tr (Y.G.); 2 Institute for Health and Sport (iHeS), Victoria University, P.O. Box 14428, Melbourne 8001, Australia; hiu.tin@live.vu.edu.au; 3 Sarcopenia Research Program, Australia Institute for Musculoskeletal Sciences (AIMSS), Melbourne 3021, Australia

**Keywords:** obesity, adipose tissue, exercise, diet, cytokines

## Abstract

The prevalence of obesity continues to rise worldwide despite evidence-based public health recommendations. The promise to adopt a healthy lifestyle is increasingly important for tackling this global epidemic. Calorie restriction or regular exercise or a combination of the two is accepted as an effective strategy in preventing or treating obesity. Furthermore, the benefits conferred by regular exercise to overcome obesity are attributed not only to reduced adiposity or reduced levels of circulating lipids but also to the proteins, peptides, enzymes, and metabolites that are released from contracting skeletal muscle or other organs. The secretion of these molecules called cytokines in response to exercise induces browning of white adipose tissue by increasing the expression of brown adipocyte-specific genes within the white adipose tissue, suggesting that exercise-induced cytokines may play a significant role in preventing obesity. In this review, we present research-based evidence supporting the effects of exercise and various diet interventions on preventing obesity and adipose tissue health. We also discuss the interplay between adipose tissue and the cytokines secreted from skeletal muscle and other organs that are known to affect adipose tissue and metabolism.

## 1. Introduction

According to the World Health Organization (WHO), worldwide obesity has nearly tripled since 1975 and reached a global epidemic [1,2]. In 2016, more than 1.9 billion (about 39%) adults worldwide were overweight and, among them, about 650 million (about 13%) were obese [2]. The increase in the prevalence of being overweight and obese has been attributed to an imbalance between energy intake and expenditure due to an increasingly sedentary lifestyle, and a nutritional transition to processed foods and high-calorie diets over the last 30 years [3]. Obesity is considered a multisystem chronic relapsing progressive disease process [4,5] adversely affecting almost all physiological functions of the body and leading to increased morbidity and mortality [5,6,7]. Furthermore, obesity is associated with many metabolic dysfunctions and comorbidities [8,9,10,11,12,13,14,15] that interfere with the quality of life and work productivity, and increases healthcare costs [16,17,18].

A positive association has been found between body mass index (BMI) over 24.9 kg/m² and overall mortality [6,7]. The associations are stronger at younger ages compared to older ages and the hazard ratio is greater in men than women [6,7]. Additionally, a population-based cohort study of 3.6 million adults in the UK revealed that life expectancy from age 40 years was 4.2 and 3.5 years shorter in men and women with obesity (BMI ≥ 30.0 kg/m²), respectively, than individuals with healthy weight (BMI 18.5–24.9 kg/m²) [7]. A recent study by Dai et al. [19] revealed that, in 2017, high BMI caused 2.4 million deaths and 70.7 disability-adjusted life years (DALYs) in females, and 2.3 million deaths and 77.0 million DALYs in males globally, based on the data from 195 countries and territories. The study showed that, although the age-standardized rate of high-BMI-related DALYs increased by only 12.7% for females and 26.8% for males, the global number of high-BMI-related DALYs has more than doubled for both sexes between 1990 and 2017 [19]. Cardiovascular disease was the leading cause of high-BMI-related DALYs, followed by diabetes and kidney diseases, and neoplasms, together accounting for 89.3% of all high-BMI-related DALYs [19]. In addition to morbidity and life expectancy, obesity is a major burden on the healthcare system due to both direct and indirect costs [16].

Adipose tissue is a highly metabolically active organ that performs many functions such as lipid storage, mechanical protection, thermal insulation, immune responses, endocrine functions, and non-shivering thermogenesis [20,21]. It has a substantial capacity to control its size and function in response to several internal and external stimuli including nutritional status and temperature, accordingly. It plays an important role in the regulation of systemic nutrient and energy homeostasis [20]. Although WHO defines overweight and obesity as the abnormal or excessive fat accumulation that may impair health (WHO fact sheet 2021), BMI is preferentially used to define overweight factors (BMI ≥ 25) and obesity (BMI ≥ 30) in epidemiological studies. However, BMI is not sensitive enough to differentiate the level or the distribution of adipose tissue mass. Furthermore, the percent of body fat (BF) for any given BMI value varies greatly among individuals based on age, sex, and ethnicity. In addition, for any given amount of BF, greater cardiometabolic risk has been associated with the localization of excess fat in the visceral adipose tissue (VAT) and ectopic depots (such as muscle, liver, and pancreas) [1,22]. Furthermore, the balance of hypertrophic expansion of existing adipocytes and adipogenesis within an individual profoundly affects metabolic health. Partly due to hypoxia and mechanical stress, large adipocytes are associated with impaired metabolic health while small adipocytes are associated with a reduced risk of metabolic decline [23]. Compared to small adipocytes, increased lipolysis and inflammatory cytokine secretion, and reduced secretion of anti-inflammatory adipokines have been observed in hypertrophic adipocytes [23]. Moreover, a subgroup of individuals with obesity, named metabolically healthy obese (MHO), is protected against cardiometabolic disturbances as compared to individuals with metabolically unhealthy obesity (MUO) [24,25], suggesting that adipose tissue distribution and dysfunction, rather than the amount of fat mass, are the crucial factors in the pathophysiology of obesity-related metabolic and cardiovascular diseases [24,25,26]. Furthermore, lower subcutaneous fat mass, adipocyte hypertrophy, and an impaired fat storage capacity of adipose tissue are the common features of MUO individuals [24,25], which may lead to ectopic fat deposition and inflammation in VAT [24,25]. On the other hand, MHO, which is more common among young, physically active individuals with a better nutritional status, is characterized by a lower degree of systemic inflammation and a favourable immune and liver function profile [24,25].

Given the underlying reasons for the dramatic increase in the prevalence of being overweight and obese during the last 40 years, population-based preventive strategies that improve social and physical environmental contexts for healthy eating and physical activity (PA) have been suggested. These preventive strategies require a multisectoral joint effort, including policymakers, educators, health professionals, food producers, city planners, etc. [27]. As a complex chronic disease, the management of obesity requires a holistic approach. Although pharmacotherapy and bariatric surgery are indicated with severe obesity, diet, exercise, and cognitive behavioural therapy are the primary strategies for the lifelong management of obesity [5,28,29]. Kheniser et al. [29] stated that two years of lifestyle interventions can facilitate a 5% weight loss and that, although a weight regain occurs, both diet and exercise interventions have substantial effects on obesity-associated comorbidities [5,29] and adipose tissue remodelling [30]. Moreover, both regular aerobic exercise and the consumption of a hypocaloric diet are associated with a substantial reduction in VAT and liver fat independent of age, biological sex, or ethnicity [31,32]. Although diet is more effective in reducing total body weight (BW) loss, exercise is superior at reducing VAT [32]. Furthermore, it has been reported that moderate-to-vigorous intensity of regular exercise for 4 to 6 months combined with a balanced, healthful diet resulted in a substantial decrease in VAT (15–20%) and that 5% to 10% of weight loss can be achieved with reasonable reductions in caloric intake with or without exercise [31]. Additionally, several organs secrete biochemicals in response to low caloric intake and exercise as well as several other factors, which contributes to the browning of white adipose tissue (WAT), and is, therefore, considered a potential therapeutic approach against obesity and associated metabolic dysfunctions [33]. Therefore, diet and exercise are the key components of weight loss and maintenance program.

Over the last 20 years, molecules secreted from skeletal muscle and other organs have been the focus of much research in terms of their therapeutic role as circulatory factors with effects on metabolically active tissue and organs. Some of these molecules released in response to muscle contraction have been reported to mediate some of the beneficial effects of exercise in other organs, such as the liver and the adipose tissue [34], such as browning of WAT and increasing thermogenesis and energy expenditure (EE), which make cytokines appealing therapeutic targets for metabolic diseases.

In this review, we provide an overview of the research-based evidence supporting the effects of exercise and various diet interventions on preventing obesity and adipose tissue health. The interplay between adipose tissue and the cytokines secreted from skeletal muscle and other organs that are known to affect adipose tissue was also discussed.

## 2. Adipose Tissue Biology: Why Our Body Is a Fat-Storing Machine?

Adipose tissue is a connective tissue mainly composed of lipid-rich cells named adipocytes [35]. It has long been believed that adipose tissue’s main function is to store energy as triglycerides while energy excess, which can then be broken down into free fatty acid and glycerol during starvation or fasting [35,36]. Since the body has a limited capacity to store glycogen, long-term imbalances between energy intake, and EE lead to a substantial increase in the amount of triacylglycerol stored in adipocytes, causing obesity [37]. Recent research has unveiled that adipose tissue also functions as an endocrine organ [38,39], which expresses and secretes factors called adipocytokines or adipokines [37,38]. Adipose tissue is a complex and essential tissue as demonstrated by the adverse metabolic consequences resulting from either excessive or deficient adipose tissue [38]. An excess of adipose tissue leads to the development of obesity and metabolic syndrome, while adipose tissue deficiency (lipodystrophy) can also cause a metabolic syndrome [40]. There are two main types of adipose tissue: WAT and brown adipose tissue (BAT). These are briefly outlined in the following section.

### 2.1. White Adipose Tissue

WAT generally stores excess energy in the form of triglycerides and makes up the majority of the human BF percentage (BF%) [41,42]. Additionally, the main functions of WAT are to protect organs against mechanical damage and release adipokines regulating various biological processes, including inflammatory reactions [43]. Adipose tissue accumulation around the abdominal cavity and mediastinum is referred to as VAT, whereas it can also be found in the hypodermis layer as subcutaneous adipose tissue (SAT) [44]. At a molecular level, WAT takes the form of single lipid droplets and has a limited number of mitochondria. WAT is not a static form of connective tissue, as it regularly remodels and changes its number of adipocytes depending on nutritional availability as well as hormonal signals [44]. Additionally, WAT is an endocrine organ capable of actively secreting free fatty acids and adipocytokines, which have autocrine, paracrine, and endocrine effects on other organs, such as skeletal muscles, the brain, and the liver [42]. Concretely, WAT is essential for energy homeostasis and metabolic regulation [35].

### 2.2. Brown and Beige Adipose Tissue

BAT, on the other hand, is mainly utilized for insulation against a cold climate. It achieves this by generating heat as a result of dissipating energy [41]. BAT is characterized by a high abundance of uncoupling protein 1 (UCP1), which is the protein responsible for non-shivering thermogenesis, along with many other genes including cell death-inducing DNA fragmentation factor-like effector A (CIDEA), PR domain containing 16 (Prdm16), and Type 2 Deiodinase (DIO2), which are all important in mitochondrial biogenesis [45]. BAT is also capable of mediating adaptive thermogenesis and, thus, contributes to the maintenance of body temperature. The role of BAT in adults has yet to be fully realized, with some studies suggesting that it may play a key role in energy homeostasis. Generally, as the BW increases with age, the amount of total BAT decreases, showing an inverse relationship between BAT and BW [41]. BAT can be found in small amounts in the neck, supraclavicular, axillar, paravertebral, perirenal/adrenal, and para-ventral regions, as well as the major vessels surrounding the heart [42]. Some studies have found that BAT can also reside in skeletal muscle tissues and even WAT [42]. At a molecular level, BAT takes the form of multiple small vacuoles and has large amounts of mitochondria [41].

More recently, another unique type of adipose tissue, beige adipose tissue, has been identified in rodents and humans [46]. Beige adipocytes are found within the WAT depots, but with similar morphology to brown adipocytes and large amounts of mitochondria [47]. In rodents, beige adipocytes can be induced by cold-exposure [48], exercise [49,50], and hypothalamic brain-derived neurotrophic factor (BDNF) [51]. To a smaller extent, beige adipocytes have been observed in humans [46], which is known to be induced by chronic exposure to peroxisome proliferator-activated receptor gamma (PPARγ) agonists [52].

## 3. Exercise Strategies to Prevent Obesity and Improve Adipose Tissue Health

Exercise plays an important role in human health as a non-pharmacological elixir that helps prevent obesity by increasing EE for weight loss, lower metabolic risk factors, and enhance adipose tissue health [53,54]. Exercise can be sub-categorised into two types: acute and chronic/training [55]. Acute exercise refers to one session of PA, while chronic/training includes repeated exercise sessions weekly or monthly [55]. Acute exercise and chronic training studies can demonstrate the short-term and long-term effects of exercise on the human body, respectively. Moreover, acute exercise interventions have been used to study the mechanistic adaptations to exercise. For instance, an acute bout of exercise increases blood flow through adipose tissue and fat mobilization, leading to the delivery of fatty acids to skeletal muscles, which is mainly based on the exercise intensity and metabolic requirements [37]. Furthermore, following an acute exercise, dietary fat stored in adipose tissue decreases as a result of the mobilization of fatty acids stimulated by β-adrenergic activation from adipose tissue to other tissues, such as skeletal muscle [37]. In addition, regular exercise/training is known to alter adipose tissue physiology, which results in enhanced fat mobilization during acute exercise [37]. It is, however, not fully elucidated whether the structural changes in adipose tissue are induced by exercise training or negative energy balance [37,45,56] and remain an important area of investigation. The relationship between PA and adiposity has been comprehensively investigated in longitudinal cohort studies that have documented a strong inverse association between measures of PA and measures of fat mass and distribution [57,58]. Thus, incorporating well-designed exercise training routines into a weight loss program are efficient strategies. In the following section, the effects of different types of exercise models on obesity and adipose tissue and the underlying molecular mechanisms are reviewed. The exercise studies included in the following sections are described in greater detail in Table 1.

### 3.1. Continuous Exercise and Adipose Tissue

Over the last 20 years, the effects of regular endurance training on adipose tissue have gained momentum and have been comprehensively investigated in numerous studies. In light of the findings of these studies, it is accepted that exercise seems to reduce fat mass, which significantly depends on the net energy deficit induced by exercise interventions. It is also noteworthy that the effect of PA without a calorie restriction diet on fat loss might be relatively small or modest [59,60]. Furthermore, a substantial energy deficit created by increased PA results in a loss of fat mass from significant depots, such as SAT and VAT. For example, an increase of daily steps from 7013 to 8840 decreased SAT and VAT as well as BF% in men with obesity [61]. Similarly, one year of training at 58% of maximal oxygen consumption (VO2_max_) (6 sessions/week) reduced total fat mass as well as abdominal visceral and SAT in nonobese women and men [60]. An experiment consisting of moderate to high-intensity aerobic training (3 sessions/week, 40 min/session, total distance 12 miles/week at 75% VO2_max_) for 8 to 9 months in individuals who are overweight and obese have reported a significant reduction in thigh SAT in both men and women who are overweight, but VAT decreased only in men [62].

Furthermore, it is well known that training studies with shorter durations exert profound effects on adipose tissue as well. For example, 24 weeks of moderate-intensity training at a low amount (LAMI, 5 days/week, 31 min/session, 50% VO2_max_) resulted in a decrease of waist circumference, similar to the moderate-intensity high amount (HAMI, 58 min/session) and high-intensity high amount (HAHI, 40 min/session, 75% VO2_max_) [63]. A follow-up study has confirmed similar reductions in total BF, SAT, and VAT among the three training groups [64]. However, there was an individual response to training in total and abdominal fat with a greater proportion of subjects “very likely” to decrease fat in the HAMI (total BF) and HAHI groups (total BF and SAT) [64]. Wilmore et al. determined the extent of changes in SAT and VAT in subjects who are overweight after 20 weeks of chronic training (3 sessions/week, at 50–75% VO2_max_ for 30–50 min) [65]. The findings implied that males had a greater loss in abdominal SAT and VAT than females. A higher rate of decline was also seen in abdominal SAT compared to VAT [65]. A study investigated 16 weeks moderate-intensity (≤lactate threshold) and high-intensity (>lactate threshold) training in women with metabolic syndrome, but no significant changes of SAT and VAT were observed in the moderate-intensity group [66]. The high-intensity group resulted in reduced total abdominal fat, subcutaneous abdominal fat, and abdominal visceral fat [66], showing that high-intensity exercise is more effective than moderate-intensity exercise training in reducing fat storage in women with obesity and metabolic syndrome.

Furthermore, research has investigated the differences between 12 weeks of moderate-intensity (4 to 5 sessions/week, EE of 1000 kcal/week, 50% VO2_max_) and high-intensity (4 to 5 sessions/week, EE of 1000 kcal/week, 75% VO2_max_) exercise on regional fat distribution in elderly adults who are overweight [67]. The findings showed a remarkable reduction in VAT in the high-intensity group exhibited, while no change was observed in the moderate group [67]. A recent study reported that vigorous-intensity physical activities are associated with high BAT density in humans, suggesting that long-term high-intensity physical activities might positively influence BAT content [68]. Collectively, the intensity of exercise training seems to play an essential role in changing adipose tissue. Another study that aimed at revealing the effect of training on adiposity in children with obesity reported a significant decrease in BF%, total BF, and SAT mass [69], following 4 months of moderate intensity training (5 sessions/week, 40 min/day at 70–75% maximal heart rate (HR_max_), equivalent to 58–66% VO2_max_). Similarly, with the diet controlled, 12 weeks of daily exercise (brisk walking or light jogging no more than 70% VO2_max_) resulted in a decrease in both SAT and VAT in men with obesity. The reduction in total fat was greater in the training group when compared with the diet-induced weight loss group (22% decrease in energy intake) [59]. Even in the exercise without a weight loss group (with 23% increase in energy intake), there was a decrease in abdominal adipose tissue and VAT [59]. Even without changes in total BW, 13 weeks of moderate-intensity training (5 sessions/week, 60 min/session, −60% peak oxygen uptake (VO2_peak_)) led to significant reductions in total, abdominal subcutaneous factors, and visceral fat in men with obesity with and without type 2 diabetes (T2D), as well as in the lean control group [70]. The reduction in VAT was greater in the groups with obesity and T2D when compared with the lean group [70]. Only 8 weeks of training at moderate intensities (70% VO2_peak_) reduced liver fat and VAT [71]. Interestingly, 45 min of training at 50% VO2_max_ (three sessions/week) seems to be enough for these reductions, as an increase of volume (to 60 min/session, 4 sessions/week) or intensity (to 70% VO2_max_) did not result in further reductions [71]. Moreover, Christiansen and colleagues compared the independent and combined effects of 12 weeks of regular exercise and diet-induced weight loss on BF distribution in subjects with obesity [72]. They reported that there was a significant decrease in BW (3.5 kg) and VAT (18%) [72]. More importantly, a hypocaloric-diet and exercise resulted in a markedly higher reduction in VAT (30–37%) and BW (12.3 kg) [72], compared to the exercise group, showing a hypocaloric diet to be more effective in reducing the VAT depot, compared to exercise only. Walhin et al. reported that 3 weeks (5 sessions/week) of moderate-intensity (50% VO2_max_) and vigorous-intensity exercise training (70% VO2_max_) combined with caloric restriction (5000 kcal/week) led to similar reductions in total fat and abdominal fat mass [73]. In addition, both exercise interventions with simultaneous restricted energy intake similarly affected the expression of the lipogenic enzymes [73].

In summary, regular exercise, especially moderate to high-intensity exercise for eight weeks to one year, decreases total BF, SAT, and VAT. Furthermore, exercise training combined with a hypocaloric-diet is more effective compared to exercise intervention alone in preventing and reducing BF.

#### Molecular Mechanisms Underlying the Reduction in Total BF, SAT, and VAT with Exercise

In vivo studies and adipose tissue biopsies following acute and chronic exercise trials have provided mechanistic insight into the molecular mechanisms that are responsible for the reduction in total BF, SAT, and VAT in response to exercise training. For example, a single session of 30 min of continuous running at 65% VO2_max_ was reported to increase whole-body fat oxidation during the post-exercise recovery period in young men [74]. Acute moderate-intensity continuous exercise at 45–70% VO2_max_ increased the oxidation of total lipid and plasma fatty acid (~60%) [75] and the amount of the adipose tissue lipoprotein lipase (56%) in men [76] but not women, and increased the number of the adipose tissue progenitor cell phenotype in adults with obesity [77]. Similarly, one hour of acute exercise at 55% VO2_max_ has been shown to modify adipose tissue mRNA and interstitial cytokine concentration in males who are overweight [78]. In addition, an increased concentration of interstitial adiponectin and interleukin (IL)-6 was detected [78], while the response at the mRNA level was different, with IL-6 mRNA increasing but adiponectin mRNA decreasing [78]. Another similar study reported increased SAT mRNA expression of vascular endothelial growth factor A (VEGFA), which is an important regulator of angiogenesis and capillary growth, in adults who are overweight/obese following acute moderate-intensity exercise at 65% VO2_max_ [79]. Furthermore, a decrease of preadipocyte content was shown in the stromal vascular cells fraction of SAT twelve hours after sixty minutes of moderate-intensity endurance exercise in adults with obesity [77]. It was also reported that a single session of 15 min exercise at 80% VO2_max_ has induced more than 3800 genes in adipose tissue from individuals who are or are not overweight, among them are the genes responsible for monocyte infiltration [80].

There are limited long-term training studies that have investigated the effects of exercise training on molecular mechanisms involved in exercise-induced changes in adipose tissue biology. One of these studies aimed to reveal gene expression changes in adipose tissue following 6 months of diet-induced and/or exercise-induced weight loss in postmenopausal women who are overweight/obese [81]. The authors showed that the mRNA expression of candidate genes in the SAT did not change in the intervention groups [81]. On the other hand, those participants with greater weight loss showed decreased expression of the leptin gene [81]. Finally, microarray analyses revealed the association of weight loss with adipose tissue gene expression involved in the synthesis of sex hormones in adipose tissue, whereas there was no impact of weight reduction with diet or diet plus exercise on genes related to inflammation in SAT in obese people [81], indicating that changes in energy balance following diet and/or exercise factors can have a limited impact on adipose tissue inflammation [82]. This field remains a fertile area of research in the near future. Furthermore, 12 weeks of endurance training (2 sessions/week supervised, 3 times/week home-based exercise at 50% VO2_max_) did not change genes involved in the control of SAT lipolysis [83] or gene expression of adipocytokines in women with obesity [84], while a decrease of plasma leptin was detected [84]. Eight weeks of exercise training (3 sessions/week, 30 min/session at 70% VO2_max_) reduced adipose tissue IL-18 mRNA content by 20% in obese individuals [85]. In addition, the mRNA expression of adipose tissue adiponectin and adiponectin receptors increased significantly after 12 weeks of training (3 sessions/week, 60 to 75 min/session at 70% of heart rate reserve) in obese men and women [86].

Findings on the browning of WAT in response to exercise come from both rodent and human studies. As reviewed comprehensively by Stanford and Goodyear [87] in rodents, it is well documented that exercise training can induce browning of WAT and the recruitment of brown-like adipocytes within WAT via exercise-induced cytokines such as irisin and IL-6, which triggered the interest in investigating WAT browning in humans. Current evidence has shown that eleven days of voluntary running resulted in increased expression of many beige adipocyte marker genes in rodent SAT [49]. Moreover, 30 days of swimming (90 min daily) increased expression of UCP1 and Prdm16 in mice SAT, suggesting browning of SAT by training in rodents [88]. While evidence from rodents seems promising, the reports of human studies are not very conclusive. First, it seems that the existence of brown/beige adipose tissue in adult humans is not very common and decreases with age [89]. Second, it was shown that endurance-trained athletes had a lower metabolic activity of BAT compared to lean sedentary individuals [90]. Furthermore, gene expression of classical brown and beige adipocyte markers in subcutaneous WAT, plasma irisin, and Il-6 levels during mild cold exposure were similar in trained and sedentary individuals [90]. Conversely, 12 weeks of cycling (3 sessions/week, 60 min/session at 43% to 70% VO2_max_) induced the mRNA expression of beige/BAT markers of UCP1, T-box transcription factor 1 (TBX1), and carnitine palmitoyltransferase-1B (CPT1B) in SAT of sedentary subjects, suggesting browning of SAT by training [91].

In summary, acute exercise interventions have shown that low-moderate-intensity exercise can increase whole-body fat oxidation, possibly by regulating adipose tissue lipolysis, gene expression of adipocytokines, or changing the cell composition of adipose tissue. However, exercise intervention may not be associated with brown and beige adipocyte recruitment in humans. Rather, endurance training can lead to the lower metabolic activity of BAT in humans. More work is needed to reveal whether particular groups or populations experience beneficial changes in adipose tissue from exercise training.

### 3.2. High-Intensity Interval Training and Adipose Tissue

For the management of obesity, it is recommended to be physically active such as 150 to 250 min/week or up to 60 min/day [92]. However, current epidemiological data indicate that the majority of the adult population does not meet the recommended PA guidelines mainly due to lack of time [93], and there is, therefore, a need to establish the efficacy of time-efficient doses of exercise that overcome the health risks associated with obesity with less time commitment. High-intensity interval training (HIIT) is characterized as a short period that must be performed over the lactate threshold, near VO2_max_, and interspersed with light exercise or rest so that extra high-intensity bouts can be performed [53,55]. HIIT is based on the Wingate test, which consists of “supra-maximal power output” [94]. A typical HIIT protocol is considered as sprint interval training (SIT), in which individuals will have to complete “all-out” several times (≥100% maximal workload capacity) performance with recovery time in between the intensive exercise sessions on a cycle ergometer [55]. Therefore, a customized low-volume HIIT protocol (near the maximal corresponding to ≥75% to <100% of maximal workload capacity effort) has been widely utilized by ample studies [95,96] and is prone to be more workable for individuals than the Wingate-based HIIT model [55].

There is robust evidence that HIIT can reduce adiposity and abdominal visceral fat despite the discrepancies available in the previous studies that are attributed to training protocols, exercise protocol, obesity status, and gender. For example, a study compared the impacts of 12 weeks (3 to 4 sessions/week) of prolonged moderate-intensity continuous training (MICT 60% VO2_max_) with HIIT (90% VO2_max_, repeated 4 min bout with 3 min recovery) on abdominal adipose tissue reduction in young women with obesity [97]. The findings showed a similar reduction in abdominal SAT and VAT in both groups [97]. Ten weeks of endurance exercise training (a combination of continuous and HIIT) improved adipose tissue insulin sensitivity. However, changes in adipose tissue composition was not reported [98]. Six weeks of HIIT (3 sessions/week, 7 × 1 min at 95–100% VO2_max_, with 1 min recovery), which resulted in increased skeletal muscle mitochondrial respiratory capacity, did not change BF% and reduced the mitochondrial respiratory capacity in SAT in overweight subjects [99]. Another study by Leggate et al. examined two weeks of HIIT (3 sessions/week, 10 × 4 min at 85% VO2_max_, 2 min rest) in sedentary males with overweight/obesity, and they found a decrease in waist circumference, as well as reductions in IL6 and fatty acid synthase content in SAT biopsies [100]. A recent experiment by Islam et al. investigated the impacts of acute high-intensity interval exercise (HIIE 10 × 4 min at 90% of HR_max_, separated by 2 min recovery) on SAT and whole-body fat oxidation in women who are overweight [101]. They showed that, despite a significant increase in whole-body fat oxidation, β-adrenergic and insulin signalling in subcutaneous adnominal adipose tissue remained unchanged following acute HIIE [101], suggesting that HIIE does not alter intracellular signalling pathways controlling fat mobilization or storage in subcutaneous abdominal adipose tissue. Another study comparing the effects of 12 weeks (3 sessions/week, 6 to 10 × 60 s intervals) moderate intensity interval training (60–80% maximal workload, with 60 s of active recovery at 40 W) with HIIT (80–90% maximal workload, with 75 s active recovery at 40 W) reported an increased fat oxidation rate in sedentary women with normal weight, overweight, and obesity [102]. However, none of the training intensity affected BW, BF%, or circumferences of waist and hip [102]. A recent study by Taylor et al. compared the impacts of HIIT with MICT on VAT and liver fat reduction in patients with coronary artery disease for 4 weeks, followed by three home-based sessions/week for 11 months [103]. The authors documented that both exercise interventions reduced VAT over 3 and 12 months, while HIIT resulted in a slightly greater reduction in liver fat when compared to MICT [103]. A meta-analysis by Keating and colleagues that reviewed 28 trials with 873 participants reported that HIIT and MICT present similar benefits for eliciting small reductions in total BF [104]. In addition, it was reported that 6 weeks of SIT (3 sessions/week, 5 × 60 s at ~128% of peak power, 90 s recovery) did not alter BF% or adipose tissue mitochondrial function [105], while it resulted in a greater loss in total BF and android fat than MICT (3 sessions/week, 45–55% HR_max_, for 20–30 min) cycling in young women who are overweight [94]. Two weeks of Wingate-based SIT (3 sessions/week) significantly reduced waist and hip circumference, and increased the resting fat oxidation rate in sedentary men who are overweight/obese [106]. Another study reported two weeks of Wingate-based SIT (3 sessions/week) resulted in a similar reduction in BF%, abdominal SAT, and VAT compared to MICT (40–60 min at 60% VO2_max_) in healthy subjects with insulin resistance [107]. Furthermore, both training interventions decreased CD26 and ANGPTL4 gene expression in SAT [107]. Finally, Cooper et al. reported no significant change in FM or abdominal VAT following 12 weeks (3 sessions/week) of SIT interventions consisting of 4 to 10 × 30 s sprint efforts in men who are overweight [108], raising further questions regarding if exercise training without caloric restriction could facilitate favourable changes in body composition and abdominal VAT.

In summary, it is apparent that interval training models improve adipose tissue despite the inconsistent and controversial findings that existed. Moreover, HIIT seems to be an alternative to MICT for reducing visceral and liver fat. More work that combines HIIT with hypocaloric diets is needed. The findings of further studies can open up new time-efficient therapeutic potential in developing new strategies for the prevention and management of obesity.

### 3.3. Resistance Exercise Training and Adipose Tissue

Resistance exercise training (RT) consists of various types of physical exercise that causes the skeletal muscles to contract against an external resistance [109,110] that develops the strength and size of muscles, and increases bone mass [111,112,113]. The metabolic effects of reduced muscle mass has been reported to result in a high prevalence of obesity, insulin resistance, and T2D [114,115]. Therefore, RT and subsequent increases in muscle mass are likely to reduce metabolic disease risk factors [114,116]. Although, the aerobic exercise has traditionally been recommended for preventing and managing obesity and associated metabolic risk factors [116,117], recently, RT has also been suggested to be a feasible and efficacious alternative to aerobic exercise for weight control due to its multiple therapeutic effects [114,116]. For example, the age-related decline in resting EE is closely associated with the loss of skeletal muscle mass [118], which can be reversed by RT that leads to increased muscle mass based on the training duration and intensity. However, despite no clinically important change in resting EE following RT, maintenance of muscle mass with RT helps prevent age-associated fat mass gains by promoting an active lifestyle [119].

Several studies have reported that RT can reduce FM and VAT in men [120] and women [109,121] independent of dietary caloric restriction [122]. A study that assessed body composition in older women reported significant decreases in visceral fat after 16 weeks of RT [123]. Similarly, another study investigated the effects of 16 weeks of RT combined with diet interventions on FM and VAT in middle-aged men with obesity. The findings showed that there was a 40% reduction in visceral fat in the RT combined diet group [122]. Hunter et al. showed that 25 weeks of chronic RT resulted in an improvement in fat-free mass and a reduction in BF in older males and females [124]. There was also a substantial loss of intra-abdominal adipose tissue and abdominal SAT in women but not in men who are overweight [124]. Ku and colleagues documented that 12 weeks of RT (5 sessions/week elastic band exercise) decreased SAT, which was comparable to 12 weeks of aerobic training (5 sessions/week, walking for 60 min at moderate-intensity [3.6–5.2 metabolic equivalents]) in individuals with T2D [110]. However, only RT decreased subfascial adipose tissue at the mid-thigh level [110]. Rosety et al. highlighted 12 weeks of resistance circuit training (3 sessions/week), which resulted in a reduced thickness of epicardial adipose tissue in obese women [125]. Ross et al. reported a substantial similar decrease in the volume ratio of VAT to SAT after 16 weeks of RT and aerobic training (3 sessions/week) combined with caloric restriction (reduced by 1000 kcal) in obese women [126]. Moreover, within the VAT depot, a remarkable reduction was observed for both intraperitoneal and extraperitoneal adipose tissue [126]. Slentz et al. compared the effects of high-intensity aerobic training (12 miles/week at 75% VO2_max_) and RT (3 times/week, 3 sets of 8–12 repetitions/set) in adults who are overweight [127]. They reported high-intensity training provided a greater reduction in VAT and total abdominal fat than RT [127], indicating high-intensity aerobic exercise to be a more effective exercise mode to reduce visceral fat.

The effects of acute resistance exercise (RE) on adipose tissue have also been investigated by a limited number of studies that documented a transient increase in adipose tissue lipolysis. For example, one study with trained men reported that acute RE (3 sets of 10 repetitions with a load at 85–100% of the individual’s 1 maximum repetition (1RM), 90 sec rest periods between all sets and exercises, for a total of 40–45 min) increased SAT lipolysis during RE, while SAT lipolysis and whole-body fat oxidation were higher immediately post RE [128]. Another acute RE (one set of 10 repetitions at 40% 1RM and three sets of 10 repetitions at 65% 1RM) study in trained women reported an increase in post-exercise whole-body fat oxidation and SAT lipolysis [129]. Chatzinikolaou et al. investigated the effect of performing 30 min of acute circuit RE (3 cycles on 10 machines selected to stress the major muscle groups, 10–12 repetitions/set at 70–75% of 1RM with 30 s rests between sets, and 2 min rests between cycles) on adipose tissue lipolysis in lean men and men with obesity [130]. The authors documented that adipose tissue triacylglycerol lipase activity was elevated by 18-fold after 5 min of exercise in lean subjects, whereas a 16-fold increase was observed 10 min after exercise in males with obesity [130]. In summary, the overall available body of literature indicates that RT with or without diet modification is an effective way to reduce BF and control obesity.

### 3.4. Concurrent Training and Adipose Tissue

Concurrent training (CT) is a designed exercise model involving aerobic and anaerobic metabolic pathways so that it can enhance the effects of both aerobic and RT models [131,132,133]. Although CT has been used among athletes for multiple decades to enhance performance in a variety of sports, it has recently grown in popularity [134,135]. As a combined form of endurance and strength exercise modes, CT induces changes in the cardiovascular and the neuromuscular systems, providing widespread disturbances occurring in local and systemic homeostasis that, in turn, results in remarkable adaptation in human physiology. In addition to providing traditional physiological adaptations known to be induced by traditional endurance exercise, CT can also improve body composition and health-related outcomes [132].

The effect of CT on FM and adiposity has been addressed in a variety of studies that have yielded contrasting results. These discrepancies may be partially due to potential factors known to alter one’s energy balance, such as caloric intake or EE, which were not usually considered in previous studies. Furthermore, some studies have reported a similar improvement in adiposity following CT or aerobic exercise [127,136,137], whereas other studies documented that CT elicited greater improvement [138]. For example, a one-year intervention (3 sessions/week) of aerobic (30 min of aerobic exercise at 50–70% VO2_max_) plus RT (30 min of RT) induced higher changes in body composition, waist circumference, and BF in adolescents with obesity rather than aerobic exercise by itself [139]. Similarly, Dâmaso and colleagues compared the effect of aerobic exercise alone or aerobic plus RT on visceral fat and its role on pro-inflammatory/anti-inflammatory adipokines in obese adolescents [138]. They reported that aerobic plus RT provided a greater reduction in visceral fat and pro-inflammatory adipokines than an aerobic training alone intervention [138], showing CT to be a more effective strategy to control central obesity in adolescents. Slentz et al. reported similar significant reductions in VAT, SAT, and total abdominal fat following aerobic plus RT (3 sessions/week, 12 miles/week at 75% VO2_max_ plus 3 × 8–12 repetitions/set, 3 sessions/week) and aerobic training alone (3 sessions/week, 12 miles/week at 75% VO2_max_) in overweight adults [127]. Similarly, Monteiro et al. reported a significant reduction in waist circumferences and BF% after 20 weeks of CT (3 times/week, 60 min at 50% of 1RM, followed by 30 min at between 65% and 85% VO2_max_ aerobic training) and aerobic training (3 times/week, 50 min of continuous exercise between 65% and 85% VO2_max_) [136]. Another study reported a significant reduction of waist circumference (~3%), VAT (~10%), and SAT (~10%) in obese adolescents following 16 weeks of CT (twice/week, 30–45 min/session 70–85% HR_max_ plus 30–45 min, 12–14 repetitions, low-heavy weights) [140]. Conversely, Norheim et al. investigated the effect of 12 weeks of CT on human abdominal subcutaneous fat in adults with normal weight and overweight [141]. The CT program consisted of two aerobic exercise sessions plus two RT exercise sessions per week. The obtained findings following the training program showed that there was no significant change in the mRNA level of PPARγ coactivator-1α (PGC-1α) of SAT, the brown-fat-selective gene Prdm16, or other known browning genes TBX1, transmembrane protein 26 (TMEM26), or tumor necrosis factor receptor superfamily member 9 (CD137) [141]. Stinkens et al. reported similar findings showing that 12 weeks of the CT program did not change abdominal subcutaneous adipocyte size, β2-adrenergic sensitivity of lipolysis, and adipose tissue gene expression of markers involved in browning and lipolysis in obese subjects [142]. Collectively, 12 weeks of CT does not seem to provide enough stimulus to induce adipocyte morphology and adipose tissue gene/protein expression in humans [142].

Taken together, it is evident that CT is a preventative and therapeutic exercise model capable of inducing similar or even superior improvement in adipose tissue and obesity to traditional endurance exercise. Given that long-term CT increases fat-free mass that results in a reduction of BF% independent of changes in fat stores, CT can be regarded as an alternative exercise mode able to decrease BF%. Health authorities should be encouraged to recommend the incorporation of CT into exercise routines. Furthermore, the effect of CT on adipose tissue morphology remains equivocal and awaits determination in further studies.

**Table 1:** Description of exercise studies that are presented in the exercise section.

**Continuous Exercise and Adipose Tissue**								
	**Author**	**Year**	**Participants** **( $\dot{V}$ O2_max_) (mL/kg/min)**	**n (M/F)**	**Duration,** **Frequency, Mode**	**Protocols**	**Main Findings**	**Ref**
1	Ross et al.	2000	Obese males (NR)	(52/0)	12 weeks, daily, brisk walking or light jogging	Group 1: Diet (reducing total daily energy intake to 700 kcal/day) Group 2: Exercise (80% of HRmax until 700 kcal is expended) Group 3: Exercise without weight loss (enough calories given to compensate for the energy expended during the daily exercise sessions) Group 4: Control group	Reduction in total fat was greater in group 2 compared with group 1. Group 2: Substantial decreased in both SAT and VAT Group 3: Attenuation in abdominal fat and prevented further weight gain.	[59]
2	Miyatake et al.	2002	Obese males (NR)	(31/0)	1 year follow up study, daily, walking	An increase of daily steps from 7013 to 8840	Significantly decreased in SAT, VAT, and body composition.	[61]
3	Racette et al.	2006	Healthy, non-obese adults (NR)	(18/30)	1 year, 6 days/wk, running/cycling/rowing ergometers/elliptical machines/stairclimbers	Group 1: 20% calorically-restricted diet Group 2: Training at 58% of VO2max Group 3: Healthy lifestyle control group	Significant reduction in fat mass, SAT, and VAT for both group 1 and 2.	[60]
4	Durheim et al.	2008	Sedentary, dyslipidemic, overweight males (~32.8 VO2peak) females (~23.9 VO2peak)	(40/33)	8–9 months, 3 days/wk, aerobic training	Group 1: ∼20 miles/wk of jogging (65–80% VO2_max_) Group 2: 12 miles/wk of jogging (65%-80% VO2_max_) Group 3: 12 miles/wk of brisk walking (40–55% VO2_max_)	Significantly reduced in thigh SAT for all three groups, but VAT decreased substantially in men only.	[62]
5	Ross et al.	2015	Abdominally obese adults (NR)	(104/196)	24 weeks, 5 days/wk, walking/jogging/treadmill training	Group 1: Training at a low-amount, moderate-intensity exercise at 50% VO2max (31 min/session) Group 2: Training at a high-amount, moderate-intensity exercise at 50% VO2max (58 min/session) Group 3: Training at a high-amount, high-intensity exercise at 75% VO2max (40-min/session) Group 4: Control group	Similar reductions were resulted in total BF, SAT, and VAT in all training groups.	[63]
6	Wilmore et al.	1999	Overweight adults (NR)	(258/299)	20 weeks, 3 days/wk, cycling	Training at 55% VO2max to at 75% VO2max for 30 min to 50 min.	Males had a greater loss in abdominal SAT and VAT than females. A higher rate of decline was also seen in abdominal SAT compared to VAT.	[65]
7	Irving et al.	2008	Middle-aged obese women (~21 VO2peak)	(0/27)	16 weeks, 5 days/wk, aerobic training	Group 1: Moderate-intensity training (5 days per week at an intensity ≤ LT Group 2: High-intensity training (3 days per week at an intensity > LT and 2 days per week ≤ LT) Group 3: No-exercise training	No significant changes of SAT and VAT were observed in group 1, whereas group 2 resulted in reduced total abdominal fat, SAT, and VAT.	[66]
8	Coker et al.	2009	Overweight elderly adults (NR)	(9/9)	12 weeks, 4–5 days/wk, aerobic training	Group 1: Moderate-intensity (50% VO2peak) Group 2: High-intensity (75% VO2peak)	A remarkable reduction in VAT in the high-intensity group exhibited, while no change was observed in the moderate group.	[67]
9	Tanaka et al.	2020	Healthy adults (NR)	(87/145)	4 months, NR, walking/aerobic training	Group 1: WM Group 2: WM + vigorous-intensity physical (VPA) activities (VWM)	VPA activities resulted in high BAT density, particularly in men. BAT-density is related to visceral fat area and VWM in men, and related to body fat percentage in women.	[68]
10	Owens et al.	1999	Obese children (NR)	(25/49)	4 months, 5 days/wk, exercising on machines and sports activities	Group 1: 40 min/day at 70–75% HRmax Group 2: Control group	Significant decrease in BF%, total BF, and SAT for group 1.	[69]
11	Lee et al.	2005	Lean and obese male with and without T2D (~61.2% VO2peak)	(24/0)	13 weeks: 5 days/wk, aerobic training	All participants trained for 60 min/day at a moderate intensity (∼60% VO2peak)	Significant reductions in total abdominal SAT and VAT in all groups (lean and obese males with and without T2D). Reduction in VAT was greater in the obese and T2D groups when compared with the lean group.	[70]
12	Keating et al.	2015	Inactive and overweight/obese adults (~22.4 VO2peak)	(17/31)	8 weeks, 3–4 days/wk, brisk walking/cycling	Group 1: Cycling and brisk walk at 50% VO2peak for 3 days and 1 day/wk, respectively. (From 45 min in week one to 60 min by the 3rd week, totaling 180–240 min/wk) Group 2: Cycling and brisk walk at 50% VO2peak for 2 days and 1 day/wk, respectively. (From 30 min in week one to 45 min by the 3rd week, totaling 90–135 min/wk) Group 3: Cycling and brisk walk at 60–70% VO2peak for 2 days and 1 day/wk, respectively. (From 30 min in week one to 45 min at 70% VO2peak by the third week, totaling 90–135 min/wk) Group 4: Control group	Reduction in liver fat and VAT for all three groups.	[71]
13	Christiansen et al.	2009	Obese adults (NR)	79	12 weeks, 3 days/wk, aerobic training	Group 1: exercise (60–75 min at 70% VO2max per training session) Group 2: hypocaloric diet (600 kcal/day) Group 3: hypocaloric diet and exercise	Reduction in BW 3.5 kg and VAT 18% in group 1. Higher reduction in BW (12.3 kg) and VAT (30–37%) in group 2 and 3 than group 1.	[72]
14	Walhin et al.	2016	Sedentary overweight men and postmenopausal women (31.5 VO2_max_)	(24/14)	3 weeks, 5 days/wk, treadmill	Group 1: Moderate intensity training (50% VO2_max_) with caloric restriction (5000 kcal/wk) Group 2: Vigorous-intensity training (70% VO2_max_) with caloric restriction (5000 kcal/wk)	Both groups resulted similar reductions in total fat and abdominal fat mass, as well as similarly affected the expression of the lipogenic enzymes.	[73]
15	Islam et al.	2018	Active young males (NR)	(8/0)	1 day, acute session, running	Group 1: 30 min continuous running at 65% VO2_max_ Group 2: 30 min of running at 85% VO2_max_ Group 3: 4 × 30 s “all-out” sprints with 4 min of rest (SIT) Group 4: No exercise	Increased whole-body fat oxidation during the post-exercise recovery period in all exercise groups and it was greatest in group 3.	[74]
16	Henderson et al.	2007	Healthy males (56.6% VO2peak) and females (48.9% VO2peak)	(10/8)	1 day, acute session, aerobic exercise	Group 1: 90 min of exercise at 45% VO2peak Group 2: 60 min of exercise at 65% VO2peak	Substantial increased for the oxidation of total lipid and plasma fatty acid in both groups. Women was more dependent on lipid during exercise, whereas during recovery, lipid metabolism is accentuated to a greater extent in men.	[75]
17	Perreault et al.	2004	Healthy lean males (59.4 VO2_max_) and females (60 VO2_max_)	(10/10)	1 day, acute session, aerobic exercise	Exercised at 85% LT for 90 min	Significantly increased the amount of the adipose tissue lipoprotein lipase (56%) in men but not women.	[76]
18	Ludzki et al.	2020	Obese adults (NR)	(3/7)	1 day, acute session, aerobic exercise	Group 1: 60 min acute session at 80% HRpeak Group 2: No acute exercise session	Increased the number of the adipose tissue progenitor cell phenotype in exercise group, as well as decreased of preadipocyte content was shown in the stromal vascular cells fraction of SAT twelve hours after exercise.	[77]
19	Hojbjerre et al.	2007	Overweight (54.6 VO2_max_) and lean males (57.1 VO2_max_)	(16/0)	1 day, acute session, aerobic exercise	Exercised for 1 h at 55% of VO2_max_	Modification of adipose tissue mRNA and interstitial cytokine concentration in overweight males. An increased concentration of interstitial adiponectin and IL-6, while the response at the mRNA level was different, with IL-6 mRNA increasing but adiponectin mRNA decreasing.	[78]
20	Van et al.	2017	Overweight and obese adults that active (51 VO2peak) and sedentary (42 VO2peak)	(8/12)	1 day, acute session, aerobic exercise	60 min of acute moderate-intensity exercise at 65% VO2_max_	Increased SAT mRNA expression of VEGFA.	[79]
21	Fabre et al.	2018	Healthy young males (46.88 VO2_max_)	(15/0)	1 day, acute session, aerobic exercise	A single session of 15 min exercise at 80% VO2_max_	Induction of more than 3800 genes in adipose tissue from lean and overweight individuals. Among them were the genes responsible for monocyte infiltration.	[80]
22	Campbell et al.	2013	Overweight/obese postmenopausal women (24.4 VO2_max_)	(0/45)	12 months, 5 days/wk, aerobic exercise	Group 1: Exercise (≥45 min of moderate-to-vigorous intensity exercise) Group 2: Diet (reducing total daily energy intake to 1200–2000 kcal/day) Group 3: Diet plus exercise Group 4: Control	Compared to the control, the mean percent BF loss was: diet, −12.6%, exercise, −3.1%, diet + exercise, −13.2%	[81]
23	Richterova et al.	2004	Obese women (NR)	(0/11)	12 weeks, 3 days/wk, home-based training	Trained at 50% VO2peak at 40 min	No changed in genes involved in the control of SAT lipolysis.	[83]
24	Polak et al.	2006	Obese sedentary premenopausal women (24.6 VO2_max_)	(0/25)	12 weeks, 5 days/wk, aerobic training/cycling	2 sessions/wk of supervised aerobic exercise (50% VO2_max_) and 3 sessions/wk of home-based exercise (cycling)	No changes of gene expression of adipocytokines in obese women, while a decrease of plasma leptin was detected.	[84]
25	Leick et al.	2007	Obese and non-obese Adults (NR)	(18/24)	8 weeks, 3 days/wk, home-based training	30 min/session at 70% VO2_max_	Reduction of adipose tissue IL-18 mRNA content by 20% in obese individuals.	[85]
26	Christiansen et al.	2010	Obese adults (NR)	(9/10)	12 weeks, 3 days/wk, home-based training	60–75 min/session at 70% 35–40% VO2_max_	Significant elevation of the mRNA expression of adipose tissue adiponectin and adiponectin receptors.	[86]
27	Stanford et al.	2015	Trained or sedentary donor mice (NR)	6	11 days, daily, running	Running daily inside the wheel cage.	Increased expression of many beige adipocyte marker genes in rodent SAT.	[49]
28	Trevellin et al.	2014	8 weeks old male mice (NR)	(36/0)	30 days, daily, swimming	90 min of swimming	Increased expression of UCP1 and Prdm16 in mice SAT.	[88]
29	Otero-Diaz et al.	2018	Non-diabetic adults (NR)	(14/19)	12 weeks, 3 days/wk, cycling	60 min/session at 43–70% VO2_max_	Induction of the mRNA expression of beige/BAT makers of UCP1, TBX1, CPT1B in SAT of sedentary subjects.	[91]
**High-Intensity Exercise and Adipose Tissue**								
	**Author**	**Year**	**Participants** **( $\dot{V}$ O2_max_)** **(mL/kg/min)**	**n (M/F)**	**Duration,** **Frequency, Mode**	**Protocols**	**Main Findings**	**Ref**
1	Higgins et al.	2016	Inactive overweight/obese young women (NR)	(0/52)	6 weeks, 3 days/wk, SIT/cycling	Group 1: SIT (30 s “all-out” sprints followed by 4 min of active recovery) Group 2: moderate-intensity continuous training (MICT) at 45–55% HRmax, for 20–30-min	SIT resulted greater loss in total BF and android fat than MICT cycling.	[94]
2	Zhang et al.	2017	Obese young women (NR)	(0/43)	12 weeks, 3–4 days/wk, cycling	Group 1: prolonged MICT 60% VO2_max_ Group 2: HIIT (90% VO2_max_, 4 min bout with 3 min recovery)	Similar reduction in abdominal SAT and VAT in both groups.	[97]
3	Riis et al.	2019	Healthy young males (43.9 VO2_max_)	(10/0)	10 weeks, 3 days/wk, cycling	The first session was 40 min at 70% VO2max, the second session 2 × 20 min at 80%−90% VO2_max_ (5 min easy biking in between), and the third session was 8 × 5 min at 90–100% (1 min easy biking in between).	Improvement in adipose tissue insulin sensitivity.	[98]
4	Dohlmann et al.	2018	Healthy sedentary adults (27 VO2_max_)	(5/7)	6 weeks, 3 days/wk, HIIT	7 × 1 min at 95–100% VO2_max_, with 1 min recovery	No change for BF% in overweight subjects, whereas the mitochondrial respiratory capacity in SAT was reduced after training.	[99]
5	Leggate et al.	2012	Overweight/obese sedentary males (NR)	(12/0)	2 weeks, 3 days/wk, HIIT	10 × 4 min at 85% VO2_max_, 2 min rest	Decreased in waist circumference, as well as reductions in IL6 and fatty acid synthase content in SAT biopsies.	[100]
6	Islam et al.	2020	Overweight women (30.3 VO2peak)	(0/10)	1 day, Acute session, HIIE	HIIE: 10 × 4 min 90% HRmax, separated by 2 min recovery	β-adrenergic and insulin signaling in subcutaneous abdominal adipose tissue remained unchanged following acute HIIE, while there was a significant decrease in the respiratory exchange ratio.	[101]
7	Astorino et al.	2013	Sedentary women (24.2 VO2_max_)	(0/23)	12 weeks, 3 days/wk, HIIT	Group 1: 6–10 × 60 s intervals at 80–90% peak power output, with 75 s recovery Group 2: 6–10 × 60 s intervals at 60–80% peak power output, with 75 or 60 s recovery	Increased fat oxidation rate in sedentary (including both normal weight to obese) women.	[102]
8	Taylor et al.	2020	Coronary artery disease patients (NR)	42	12 months, 3 days/wk for 4 weeks, followed by three home-based sessions/wk for 11 months, HIIT/MICT	HIIT: 4 × 4 min high-intensity interval training MICT: 40 min of usual care	Both exercise interventions reduced VAT over 3 and 12 months, while HIIT resulted in a slightly greater reduction in liver fat compared with MICT.	[103]
9	Larsen et al.	2015	Overweight adults (NR)	NR	6 weeks, 3 days/wk, HIIT	5 × 60 s at ~128% of peak power, 90 s recovery	No alteration in BF% or adipose tissue mitochondrial function.	[105]
10	Whyte et al.	2010	Overweight/obese sedentary men (NR)	(10/0)	2 weeks, 3 days/wk, Wingate-based SIT	4 to 6 repeats of 30 s Wingate anaerobic sprints on an electromagnetically braked cycle ergometer, with 4.5 min recovery.	Significant reduction in waist and hip circumference in overweight/obese sedentary men, as well as an elevated resting fat oxidation rate.	[106]
11	Honkala et al.	2020	Inactive, healthy adults with IR (<40 VO2peak)	(28/26)	2 weeks, 3 days/wk, Wingate-based SIT	SIT: 4–6 × 30 s at maximum effort MICT: 40–60 min at 60% VO2_max_	Both groups resulted in a similar reduction in BF%, abdominal SAT and VAT, as well as decreased CD26 and ANGPTL4 gene expression in SAT.	[107]
12	Cooper et al.	2016	Overweight men (NR)	(30/0)	12 weeks, 3 days/wk, SIT	SIT: 4–10 × 30 s sprint efforts with passive or active recovery	No significant changes in FM or abdominal VAT.	[108]
**Resistance Exercise and Adipose Tissue**								
	**Author**	**Year**	**Participants** **( $\dot{V}$ O2_max_)** **(mL/kg/min)**	**n (M/F)**	**Duration,** **Frequency, Mode**	**Protocols**	**Main Findings**	**Ref**
1	Schmitz et al.	2003	Midlife women (NR)	(0/60)	15 weeks, 2 days/wk, RT	The treatment group performed twice-weekly supervised strength training followed by 6 months of unsupervised training.	Reduction in FM and VAT.	[109]
2	Ku et al.	2010	Women with T2D (NR)	(0/44)	12 weeks, 5 days/wk, RT/aerobic training	RT: elastic band training Aerobic training: Walking for 60 min at moderate-intensity (3.6–5.2 metabolic equivalents)	RT resulted in a greater reduction in SAT than aerobic training, as well as only RT, which decreased subfascial adipose tissue at the mid-thigh level.	[110]
3	Treuth et al.	1994	Healthy men (NR)	(13/0)	16 weeks, RT	16-week strength-training program	Reduction in FM and VAT.	[120]
4	Prabhakaran et al.	1999	Healthy, sedentary, premenopausal women (NR)	(0/24)	14 weeks, 3 days/wk, RT	Group 1: 45–50 min RT sessions (85% of 1 RM) Group 2: no exercise	Reduction in FM and VAT for group 1.	[121]
5	Ross et al.	1996	Obese men (NR)	(33/0)	16 weeks, 5 days/wk, RT/RT combined with diet interventions/only diet intervention	Group 1: RT Group 2: RT combined with diet interventions Group 3: Diet intervention	RT group has shown a decrease in FM and VAT, whereas 40% reduction in visceral fat only observed in the RT combined diet group.	[122]
6	Treuth et al.	1995	Older women (NR)	(0/14)	16 weeks, 3 days/wk, RT	Strength was assessed by one-repetition maximum tests, with training intensity gradually increased to approximately 67% of one repetition maximum	Significant reduction in visceral fat.	[123]
7	Hunter et al.	2002	Older adults (NR)	(14/12)	25 weeks, 3 days/wk, RT	Training consisted of two sets of 10 repetitions at 65–80% of 1 RM	Improvement in fat-free mass and a reduction in fat mass in older males and females. Substantial loss of intra-abdominal adipose tissue (IAAT) and abdominal SAT in overweight females, but not in overweight men.	[124]
8	Rosety et al.	2015	Obese women (NR)	(0/48)	12 weeks, 3 days/wk, resistance circuit training program	This training was circularly performed in six stations: arm curl, leg extension, seated row, leg curl, triceps extension and leg press.	Reduced thickness of epicardial adipose tissue.	[125]
9	Ross and Rissanen	1994	Obese women (NR)	(0/24)	16 weeks, 3 days/wk, RT/aerobic training combined with caloric restriction	Group 1: RT Group 2: aerobic training combined with caloric restriction (reduced by 1000 kcal)	Substantial similar decrease in the volume ratio of VAT to SAT after RT and aerobic training combined with caloric restriction.	[126]
10	Slentz et al.	2011	Overweight adults (NR)	(44/56)	8–10 weeks, 3 days/wk, RT or high-intensity aerobic training	Group 1: RT (3 times/wk, 3 sets of 8–12 repetitions/set) Group 2: high-intensity aerobic training (12 miles/wk at 75% VO2_max_)	High-intensity training provided a greater reduction in VAT and total abdominal fat than RT.	[127]
11	Ormsbee et al.	2007	Trained men (NR)	(8/0)	1 day, acute session, RE	Three sets of 10 repetitions with a load at 85–100% of the individual’s one 1RM, 90 s rest periods between all sets and exercises, for a total of 40–45 min	Increased SAT lipolysis during RE, while SAT lipolysis and whole-body fat oxidation were higher immediately post RE.	[128]
12	Allman et al.	2019	Trained women (NR)	(0/13)	1 day, acute session, RE	One set of 10 repetitions at 40% 1RM and three sets of 10 repetitions at 65% 1RM	İncreased in post-exercise whole-body fat oxidation and SAT lipolysis.	[129]
13	Chatzinikolaou et al.	2008	Lean men and obese males (NR)	(17/0)	1 day, 30 min session, RE	Three cycles on 10 machines selected to stress the major muscle groups, 10–12 repetitions/set at 70–75% of one-repetition maximum with 30 s rests between sets and 2 min rests between cycles	Adipose tissue triacylglycerol lipase activity was elevated by 18-fold after 5 min of exercise in lean subjects, whereas a 16-fold increase was observed 10 min after exercise in obese males.	[130]
**Concurrent Training and Adipose Tissue**								
	**Author**	**Year**	**Participants** **( $\dot{V}$O2_max_)** **(mL/kg/min)**	**n (M/F)**	**Duration,** **Frequency, Mode**	**Protocols**	**Main Findings**	**Ref**
1	Slentz et al.	2011	Overweight adults (NR)	(41/51)	8–10 weeks, 3 days/wk, aerobic plus RT or aerobic training	Aerobic plus RT: 12 miles/wk at 75% VO2_max_ plus 3 sets of 8–12 repetitions/set High-intensity aerobic training: 12 miles/wk at 75% VO2_max_	Similar significant reductions in VAT, SAT, and total abdominal fat for both groups.	[127]
2	Monteiro et al.	2015	Obese adolescents (NR)	32	20 weeks, 3 days/wk, CT or aerobic training	CT: 60 min of 50 % of RM, followed by 30 min of 65 and 85% VO2_max_ aerobic training. Aerobic training: 50 min continuous exercise at 65–85% VO2_max_	Significant reduction in waist circumferences and BF% after CT and aerobic training.	[136]
3	Damaso et al.	2014	Obese adolescents (NR)	139	1 year, 3 days/wk, aerobic plus RT or aerobic training	Group 1: aerobic plus RT Group 2: AT	Aerobic plus RT provided a greater reduction in visceral fat and pro-inflammatory adipokines than AT alone intervention.	[138]
4	de Mello et al.	2011	Obese adolescents (NR)	(20/10)	1 year, 3 days/wk, aerobic plus RT	Aerobic (30 min of aerobic exercise at 50–70% VO2_max_) plus RT (3 sets, 6–20 repetitions, 90–45 s/exercise/session)	Induced higher changes in body composition, waist circumference, and BF in obese adolescents than aerobic exercise only.	[139]
5	Davis et al.	2011	Obese adolescents (NR)	(0/38)	16 weeks, 2 days/wk, CT	30–45 min/session 70–85% HRmax plus 30–45 min, 12–14 repetitions, low-heavy weights	Significant reduction of waist circumference (~3%), VAT (~10%), and SAT (~10%).	[140]
6	Norheim et al.	2014	Overweight males (NR)	(26/0)	12 weeks, 4 days/wk, aerobic plus RT	12 weeks of CT	Chronic training increased the mRNA level of PGC-1α of SAT by 1.2-fold and 1.6-fold in the control group and the pre-diabetes group, respectively, whereas no significant changes neither in the brown-fat-selective gene Prdm16 or other known browning genes TBX1, TMEM26, and CD137 for both groups.	[141]
7	Stinkens et al.	2018	Obese males (NR)	(21/0)	12 weeks, 3 days/wk, CT	Aerobic exercise (30 min at 70% of maximal power output) + resistance exercise (3 × 10 repetitions at 60% of 1 repeated maximum)	No significant changes in abdominal subcutaneous adipocyte size, β2-adrenergic sensitivity of lipolysis, and adipose tissue gene expression of markers involved in browning and lipolysis in obese subjects.	[142]

BAT: brown adipose tissue. BF: body fat. BW: body weight. CD137: tumor necrosis factor receptor superfamily member 9. CPT1B: carnitine palmitoyltransferase 1B. CT: concurrent training. FM: fat mass. HRmax: maximal heart rate. HRpeak: peak heart rate. HIIE: high-intensity interval exercise. HIIT: high-intensity interval training. IL-6: Interleukin 6. IL-18: Interleukin 18. LT: lactate threshold. MICT: moderate-intensity continuous training. NR: not reported. PGC-1α: peroxisome proliferator-activated receptor gamma coactivator-1-alpha. Prdm16: PR domain containing 16. RE: resistance exercise. RM: repetition maximum. RT: resistance training. 1RM: 1 maximum repetition. SAT: subcutaneous adipose tissue. SIT: sprint interval training. TBX1: T-box transcription factor 1. TMEM26: Transmembrane protein 26. T2D: type 2 diabetes. UCP 1: uncoupling protein 1. VAT: visceral adipose tissue. VEGFA: Vascular Endothelial Growth Factor A. VO2max: maximal oxygen uptake. VO2peak: peak oxygen uptake. VPA: vigorous-intensity physical activities. VWM: walking and moderate physical activity + vigorous-intensity physical activities. WM: walking and moderate physical activity.

## 4. Diet Strategies to Prevent Obesity and Improve Adipose Tissue Health

Adipose tissue is a metabolically dynamic organ that is considered not only the primary storage site for excess energy but also an endocrine organ capable of synthesizing several biologically active compounds that regulate metabolic homeostasis [143]. Excess adiposity leads to several changes in the biology, morphology, and function of adipose tissue, such as adipocyte hypertrophy and hyperplasia, adipose tissue inflammation, and fibrosis, and impaired secretion of adipokines, contributing to the onset of obesity-related comorbidities [144]. Since one of the main causes of obesity is positive energy balance in which energy intake exceeds EE, the first approach for excess BF management and obesity prevention is the implementation of a diet combined with increased PA [145]. Although the importance of energy intake and diet composition in metabolism and energy balance have been emphasized in general [146], molecular adaptation of adipose tissue and the degree of weight loss in response to a variety of diets is still a matter of debate [147].

This section summarizes the effects of various diets on adipose tissue, body composition, and metabolism under the three main headings. Manipulation of diet composition (low-carbohydrate (CHO), low-fat, high-fat, high-protein), manipulation of timing (intermittent fasting (IF): periodic fasting, alternate-day fasting, time-restricted eating), and elimination/restriction of a specific food group (plant-based diets (PBDs), Mediterranean diet). The diet studies included in the following sections are described in greater detail in Table 2.

### 4.1. Manipulation of Diet Composition

There is ample evidence that supports moderate weight loss has many beneficial health effects [148]. Even though reducing energy intake and increasing EE are widely recommended for weight loss and improving body composition, there is still a continuous debate over the optimal macronutrient composition of the diet, such as low-CHO high-fat (LCHF) diets, low-fat high-CHO (LFHC) diets, ketogenic diets (KD), and high-protein diets (HPD).

Dietary CHO and excess secretion of insulin play a major role in the accumulation of BF [149,150], which is referred to as the CHO-insulin model of obesity [151]. This model suggests that a high proportion of CHO in the diet is likely to result in increased insulin secretion, which suppresses the release of fatty acids into circulation, leading to increased fat storage [152]. Furthermore, the reduced availability of fatty acids to metabolically active tissues leads to a state of cellular starvation, possibly due to an increased ratio of cellular adenosine monophosphate to adenosine triphosphate [153], resulting in an adaptive decrease in EE and an increase in food intake [149,153]. Therefore, it is speculated that the positive energy balance associated with the development of obesity is the result of an insulin-driven shift toward fat storage and a decrease in fat oxidation due to an increased proportion of dietary CHO [152]. In this context, diets that suppress the increase in blood glucose levels after eating likely provide a metabolic advantage. Thus, one of the possible effective methods of preventing or reducing the risk of the CHO-insulin model of obesity is to reduce the CHO proportion of the diet [154]. Low-CHO diets (LCDs) for decreasing BW have been known since the 1860s [155]. LCDs are based on the assumption that decreasing dietary CHO and increasing the amount of fat may reduce insulin secretion, increase fat mobilization from adipose tissue, and stimulate the oxidation of free fatty acids [152,156]. As a result, these metabolic changes eventually can lead to a decrease in hunger as well as an increase in BF loss and EE [152]. LCDs, <26% CHO of total energy intake or <130 g CHO/day, contain an average of 20 to 120 g of CHO, which can be planned as either high protein-normal fat or a normal protein-high fat diet. A varying amount of weight loss has been reported after diets with altered macronutrients composition [152,157]. A meta-analysis that included the studies with a duration of 6 months or longer dietary intervention, investigated the effects of LCDs and low-fat diets (LFDs) on weight loss [158]. The results showed that people on LCDs experienced greater BW loss (2.17 kg). Despite the sound theory of the CHO-insulin model of obesity, conflicting results have been found in clinical trials comparing LCDs with LFDs. Studies comparing the effectiveness of these two diets documented similar weight loss in both groups [152,157,159], and even more increased BF loss was reported when dietary fat was reduced rather than CHO [160]. Moreover, a meta-analysis by Hall et al. who reviewed 32 controlled studies concluded that both EE and fat loss were greater with lower fat diets when compared with isocaloric LCDs [154]. Additionally, some systematic reviews comparing the effects of LCHF diets and LFHC diets on weight control have concluded that both diets have similar effects on weight loss [161,162,163]. The effect of LCDs on adipose tissue metabolism is still controversial. Similarly, a recent systematic review [164] and meta-analysis studies [165] have documented that, although it is biologically plausible that the ingestion of dietary components can alter human BAT activity, the current level of evidence shows human BAT activity is not significantly affected by nutrition/diet. More work is needed to understand whether dietary components can exert a profound effect on human BAT that will allow us to reveal effective diet interventions able to activate and recruit human BAT.

KD is another type of LCHF diets that involves severely limiting CHO intake while maintaining moderate protein intake and consuming a minimum of 70% of energy from healthful fats [166]. KD was introduced in the 1920s to mimic the metabolism of fasting to treat epilepsy [167] and has recently gained significant momentum as a diet manipulation model for promoting weight loss and treatment of T2D [168]. Current evidence suggests that KDs, that are considered to be a safe and effective method for weight loss and improving metabolic control [169,170,171], can lead to a decrease in CHO metabolism, an increase in lipid oxidation, and an improved conversion of free fatty acids into ketone bodies [168]. In addition, it has been reported that there might be some side effects observed following KDs, such as headache, fatigue, constipation, and muscle cramps, especially in the period of adaptation to the diet [166,172]. Furthermore, a meta-analysis that investigated the association between the percentage of energy from CHO intake and all-cause mortality has reported that both low and high percentages of CHO were associated with increased mortality, and, therefore, the authors suggested that CHO intake should be 50–55% of total energy intake for minimal risk [173].

The success of an LCD is also attributed to its high protein content, rather than low CHO content [174]. HPD is a diet that has a high-fat content and at least 20% of energy derived from protein. The contribution of dietary protein to weight loss and long-term weight maintenance is attributed to the following effects: sustainment of the feeling of satiety despite a negative energy balance, maintenance of basal EE despite BW loss, and prevention of the fat-free mass loss [174]. Furthermore, HPDs are suggested to be more effective in weight loss compared to high CHO or high fat diets due to high satiating and thermogenic effects of proteins [175,176,177,178]. In contrast, some clinical trials lasting more than one year indicated no significant difference in weight loss following HPDs [179,180]. Furthermore, HPDs often contain high amounts of animal foods and saturated fat, which can have detrimental effects on cardiovascular health.

In conclusion, although LCDs have several potential benefits for the treatment of obesity, more research is required to understand their long-term effects as well as the variable effects on the endocrine control of glucose and lipid metabolism. When evaluated in terms of HPDs, although higher protein intake seems to provide beneficial effects on weight control, there are some caveats, such as increased acid load on the kidneys or higher saturated fat content of animal proteins. More research is needed to demonstrate the long-term effects of both LCDs and HPDs.

### 4.2. Elimination/Restriction of a Specific Food Group: Plant-Based Diets

Excess BF is an important risk factor for cardiometabolic diseases and the associated mortality [181]. Dietary composition and a high level of blood triglycerides are associated with increased BF [182]. In this regard, PBDs, defined as dietary patterns that include foods of plant origin, especially vegetables, fruits, grains, and legumes, have been suggested to have beneficial effects on blood lipids and adiposity [183]. PBDs including vegan and vegetarian diets [184], Mediterranean style diet [172], and the Nordic diet [185] usually exclude or rarely include meats, but may contain dairy products, eggs, and fish. PBDs have been associated with a reduced risk for developing chronic diseases [183,186,187,188,189]. Additionally, there is an increasing number of research studies that indicates the use of PBDs as an effective dietary approach for weight loss [184]. Furthermore, the diet content of the PBDs may be of higher quality than other energy-restricted diets [183].

Several studies have reported that PBDs may lower BMI and result in an improvement in chronic diseases [183,187]. In the Adventist Health Study-2, mean BMI was found to be highest in meat-eaters and lowest in those who avoided all animal products [190]. Similarly, a recent study showed a decrease in BMI of 4.4 kg/m^2^ with a six-month of PBD with no energy restrictions, compared with usual care (0.4 kg/m^2^), in overweight or obese individuals [191]. Potential mechanisms behind this link may involve numerous biologic pathways, including changes in satiety [192] and inflammation [186]. Furthermore, a meta-analysis showed that PBDs are associated with an improvement in obesity-related inflammatory profiles and could provide a cure for therapy and prevention of chronic disease risk [186]. It is also worth noting that some of the plant-based foods include bioactive compounds, which have anti-obesity and anti-inflammatory effects [193]. A bioactive compound is a substance that has biological activity and can improve health conditions. Fruits, vegetables, nuts, seeds, and spices are rich in bioactive compounds. Two recent reviews [194,195] have reported a strong association between the health benefits of foods containing bioactive compounds and their ability to regulate gene expression in adipose tissue, based on the clinical studies, in vivo studies, and in vitro studies. Therefore, dietary interventions that have limited adverse effects and include more bioactive food compounds might be effective strategies in preventing obesity and metabolic diseases.

Compared with diets rich in animal products, PBDs contain lower amounts of total fat, saturated fat, cholesterol, and total energy, while being rich in unsaturated fatty acids and fiber [196]. Increased dietary fiber contributes to satiety by increasing the volume of food in the stomach, decreases the energy density of the diet, and, thus, results in weight loss [197]. Furthermore, the increased dietary fiber has a cholesterol-lowering effect, as soluble fibers bind bile acids in the small intestines and increase the excretion of bile salts in the feces [198]. Therefore, a high fiber consumption, accomplished with greater adherence to a PBD, has been associated with decreased bodyweight, lower blood pressure, decreased risk of T2D, and improved blood lipids [199]. Furthermore, PBDs are rich in antioxidants (especially vitamin C and E) and exert anti-inflammatory effects [200]. In addition, vegetable proteins in PBDs are known to decrease the levels of blood lipids and the risk of obesity and cardiovascular disease, and induce hepatic fatty acid oxidation [201,202].

A study investigated whether adhering to more PBD, beyond strict vegan or vegetarian diets could help prevent adiposity in a middle-aged and elderly population [203]. In this population-based cohort of middle-aged and elderly participants, a higher adherence to a more plant-based, less animal-based diet was associated with less adiposity over time, irrespective of the general healthfulness of the specific plant and animal-based foods [203]. Similarly, Ratjen et al. [204] found that adherence to PBDs was associated with lower VAT. Furthermore, the effects of PBDs may differ according to the plant-based dietary spectrum. In this respect, Turner-McGrievy et al. [205] compared the effectiveness of five different PBDs (vegan [n = 12], vegetarian [n = 13], pesco-vegetarian [n = 13], semi-vegetarian [n = 13], or omnivorous [n = 12]) for weight loss. The results showed that the weight loss in the vegan group was significantly higher than the omnivorous, semi-vegetarian, and pesco-vegetarian groups. On the other hand, restricting or eliminating a food group from the diet can result in nutrient deficiencies, especially for pregnant or lactating women, children, and adolescents [206]. Therefore, it should be considered that some nutritional deficiencies including protein, calcium, iron, and vitamin B12 may be due to PBDs [199].

In summary, PBDs appear to reduce the risk of metabolic syndrome and are associated with lower BMI, lower concentrations of triglycerides, and total and low-density lipoprotein cholesterol. Additional research that examines the effects of PBDs on adipose tissue and obesity management for longer periods are needed.

### 4.3. Manipulation of Timing: Intermittent Fasting

The negative energy balance required for weight loss may be achieved by using 20–40% daily calorie restriction [145]. In recent years, a periodic and repeated energy restriction strategy, namely IF, have become increasingly popular as an alternative weight loss strategy. IF consists of abstaining from food and caloric beverages for a certain period alternated with normal eating [207]. IF strategies may differ in length and the frequency of the fasting durations. IF can be combined with exercise interventions and other diet types. The most common types of IF include periodic fasting or 5:2 diet, alternate-day fasting, time-restricted feeding, and religious fasting [207]. The main aim of fasting is to promote changes in metabolic pathways, cellular processes, and hormones [208]. The most common physiological changes observed in response to IF include improved insulin sensitivity and reduced levels of blood pressure, BF, fasting glucose, and inflammation [207,209].

Various reviews have compared the results of IF with continuous energy restriction [210,211]. One of these reviews has reported that IF led to 3–8% reductions in BW after 3 to 24 weeks and 4–14% reductions after 6 to 24 weeks in comparison to an energy restriction regimen [210]. A study comparing the effects of IF or continuous energy restriction on weight loss and metabolic disease risk markers in young, overweight women indicated that intermittent energy restriction is an effective intervention as continuous energy restriction in reducing BW [212]. Another study that also compared the effects of intermittent and continuous energy restriction on body composition and adipose tissue gene expression over 50 weeks showed that intermittent calorie restriction may be equivalent but not superior to continuous calorie restriction for weight loss [213]. In a long-term, randomized clinical trial, consisting of 6 months of a weight loss phase and 6 months of a weight maintenance phase, Trepanowski et al. [214] compared the effects of alternate-day fasting vs. daily calorie restriction on weight loss, weight maintenance, and risk indicators for cardiovascular disease in adults with metabolically healthy obesity. Findings of the study [214] revealed that alternate-day fasting and the daily calorie restriction resulted in a similar weigh loss at month 6 (−6.8% vs. −6.8%) and at month 12 (−6.0% vs. −5.3%), and that the risk factors for cardiovascular disease including blood pressure, heart rate, triglycerides, fasting glucose, fasting insulin, insulin resistance, C-reactive protein, and homocysteine concentrations at month 6 and 12 were similar in the intervention groups. However, the dropout rate was higher in the alternate-day fasting group (38%) as compared to the daily calorie restriction group (29%). The authors [214] concluded that alternate-day fasting did not produce superior adherence, weight loss, weight maintenance, or cardio-protection vs. daily calorie restriction. Results from these intervention studies concluded that these timing manipulation patterns result in weight loss, with modest and mixed effects on glucose metabolism and lipid levels.

Furthermore, it is worth mentioning that short-term side effects of fasting are dependent on the length of the fasting period and may be similar to KD, such as fatigue, headaches, and constipation [215]. Research also shows that IF can be beneficial in eating behaviours and mood among subjects who are overweight and obese, but might have harmful effects among normal weight individuals with unrestrained eating behaviours [212,215]. It is also important to consider that IF might have harmful effects on children and elderly individuals.

In summary, there is growing evidence that shows the metabolic health benefits of IF, making IF a feasible, safe, and tolerable diet model for promoting metabolic health and weight loss. In addition, current evidence shows that IF does not lead to a higher weight loss in comparison with continuous calorie restriction regimens and there are limited data regarding other clinical outcomes, such as diabetes and cardiovascular diseases. Further research is needed to determine the long-term effects of IF regimens on health in different populations.

**Table 2:** Description of diet studies that are presented in the diet section.

**Effects of Diet Composition Manipulation on Body Weight and Health**								
	**Author**	**Year**	**Diet**	**Participants**	**n (M/F)**	**Duration**	**Main Findings**	**Ref**
1	Hall et al.	2016	Low-CHO ketogenic isocaloric diet or high-CHO diet	Overweight and obese men Mean age: 33 ± 1.8 y BMI: 28.8 ± 0.8 kg/m^2^	(17/0)	4 weeks high-CHO diet and 4 weeks ketogenic diet	Weight loss KD: 2.2 ± 0.3 kg (0.5 ± 0.2 kg from loss of body fat) BD: 0.8 ± 0.2 kg (0.5 ± 0.1 kg from loss of body fat) Increase in EEchamber, sleeping EE and EEDLW, decrease in RQ compared with baseline diet	[152]
2	Foster et al.	2010	LCD or LFD (limiting energy intake to 1200 to 1500 kcal/d for women and 1500 to 1800 kcal/d for men, 55% CHO, 30% fat, 15% protein)	Obese adults 45.5 ± 9.7 y BMI: 36.1± 3.5 kg/m^2^	(99/208)	2 years	Weight loss LCD: −6.34 kg LFD: −7.37 kg Fat mass loss LCD: −3.99 kg LFD: −3.84 kg Higher increase in HDL in LCD group Similar reductions in TG, LDL, VLDL, systolic blood pressure	[157]
3	Ebbeling et al.	2012	Isocaloric LFD (60% CHO 20% fat, 20% protein) or low-glycemic index diet (40% CHO, 40% fat, 20% protein), or VLCD (10% CHO, 60% fat, 30% protein)	Overweight and obese young adults Mean age: 30.3 ± 5.7 y BMI: 34.4 ± 4.9 kg/m^2^	(13/8)	Crossover design; 12 weeks	Highest decreases in REE and TEE with LFD. Leptin level was highest in the LFD and lowest in the VLCD. HDL was highest in VLCD and lowest in LFD.	[159]
4	Hall et al.	2015	Isocaloric reduced fat diet or reduced CHO diet	Obese adults Mean age: 35.4 ± 1.74 y BMI: 35.9 ± 1.1 kg/m^2^	(10/9)	5 to 7 weeks	Greater weight loss and increased fat oxidation in RC diet than RF diet at the 6th day and greater fat loss (463 ± 37 g) in the RF diet compared to the RC diet (245 ± 21 g).	[160]
5	Dyson et al.	2007	LCD (≤40 g CHO/day) or healthy-eating diet	Overweight or obese with T2DM or non-diabetic Mean age: 52 ± 9 y BMI: 35.1 ± 7.0 kg/m^2^	(8/18)	3 months	Weight loss LCD: −6.9 kg Healthy eating diet: −2.1 kg No difference in changes in HbA1c, ketone, or lipid levels.	[169]
6	Goday et al.	2016	Very low-calorie-ketogenic (VLCK) or low-calorie diet	Obese adults with T2DM Mean age: 54.5 ± 8.4 y BMI: 33.07 ± 1.56 kg/m^2^	(31/58)	4 months	Weight loss VLCK: −14.7 kg LC: −5 kg The reduction in HbA1c and glycemic control was greater in the VLCK group.	[170]
7	Harvey et al.	2019	VLCKD (5% CHO) or LCD (15% CHO) or moderate-low CHO diet (MCD) (25% CHO)	Healthy adults Mean age: 38.9 ± 7.1 y BMI: 27.0 ± 3.96 kg/m^2^	(14/25)	12 weeks	Weight loss VLCKD: −4.12 kg LCD: −3.93 kg MCD: −2.97 kg Similar reductions in total cholesterol, LDL, TG, and increase in HDL	[171]
8	Dalle Grave et al.	2013	HPD (34% protein, 46% CHO) or HCD (17% proteins, 64% CHO)	Obese adults Mean age: 46.7 ± 11.1 y BMI: 45.6 ± 6.7 kg/m^2^	(37/51)	1 year	Weight loss HPD: −18.1 kg (15.0%) HCD: −15.9 (13.3%) Similar reductions in TG, LDL, total cholesterol, glucose, and insulin levels and increase in HDL.	[179]
**Effects of Plant-Based Diets on Body Weight and Health**								
	**Author**	**Year**	**Diet**	**Participants**	**n (M/F)**	**Duration**	**Main Findings**	**Ref**
1	Wright et al.	2017	Low-fat plant-based diet (7–15% total energy from fat) or control	Obese, overweight, and diagnosed with at least one of T2DM, ischaemic heart disease, hypertension or hypercholesterolaemia Mean age: 56 ± 9.7 y BMI: 34.3 ± 1.9 kg/m^2^	(26/39)	6 to 12 months	Significant reduction in BMI (4.2 kg/m^2^) in diet group	[191]
2	Thompson et al.	2005	Standard diet or high-dairy diet or high-fiber and high-dairy diet	Obese adults Mean age: 41.4 ± 8.9 y BMI: 34.8 ± 3.1 kg/m^2^	(72/0)	48 weeks	Similar weight loss in all diet groups Standard diet: 10.1 kg High-dairy diet: 11.7 kg High fiber and high dairy diet: 10.4 Similar fat mass loss in all diet groups Standard diet: −7.5 kg High-dairy diet: −9.0 kg High fiber and high dairy diet: −8.5 kg Similar increase in HDL and reductions in total cholesterol, LDL, fasting glucose and insulin, leptin, hs-CRP	[197]
3	Turner-McGrievy et al.	2015	Vegan Vegetarian Pesco-vegetarian Semi-vegetarian Omnivorous	Overweight or obese adults Mean age: 48.74 ± 7.5 y BMI: 34.96 ± 5.2 kg/m^2^	(17/46)	6 months	Weight loss Vegan: −7.5% Vegetarian: −6.3% Pesco-vegetarian: −3.2% Semi-vegetarian: −3.2% Omnivorous: −3.1%	[205]
**Effects of Intermittent Fasting on Body Weight and Health**								
	**Author**	**Year**	**Diet**	**Participants**	**n (M/F)**	**Duration**	**Main Findings**	**Ref**
1	Varady et al.	2009	Alternate day fasting (Fast day: 25% of energy needs, alternated day: ad libitum food intake)	Obese adults Mean age: 46.0 ± 2.4 y BMI: 33.8 ± 1.0 kg/m^2^	(4/12)	10 weeks	Weight loss −5.6 ± 1.0 kg Body fat percent decreased from 45 ± 2% to 42 ± 2% Significant decreases in total cholesterol, LDL, TG, and blood pressure	[209]
2	Harvie et al.	2011	IER (∼2710 kJ/day for 2 days/week) or CER (∼6276 kJ/day for 7 days/week)	Overweight or obese premenopausal women Mean age: 40.05 y BMI: 30.6 ± 5.1 kg/m^2^	(0/107)	6 months	Weight loss IER: −6.4 kg CER: −5.6 kg Similar reductions in hs-CRP, leptin, total cholesterol, LDL, TG, and blood pressure. Reductions in fasting insulin and insulin resistance in both groups greater in the IER group.	[212]
3	Schübel et al.	2018	ICR (5:2, weekly energy deficit ∼20%) or CCR (daily energy deficit ∼20%) or control group	Overweight and obese adults Mean age: 50.2 ± 8 y BMI: 31.4 ± 3.8 kg/m^2^	ICR: n: 49 CCR: n: 49 Control: n:52	50 weeks	Weight loss ICR: −5.2% ± 1.2%, CCR: −4.9% ± 1.1% Control: −1.7% ± 0.8% Similar reductions LDL, HDL, cholesterol, TG, insulin, HOMA-IR levels, and adipokines (adiponectin, leptin)	[213]

BMI, body mass index. CER, continuous energy restriction. CCR, continuous calorie restriction. CHO, carbohydrate. EE, energy expenditure. EEDLW, energy expenditure measured by doubly labeled water. HCD, high-carbohydrate diet. HDL, high-density lipoprotein. hs-CRP, high sensitivity C-reactive protein. HPD, high-protein diet. ICR, intermittent calorie restriction. IER, intermittent energy restriction. LCD, low-carbohydrate diet. LDL, low-density lipoprotein. LFD, low-fat diet. REE, resting energy expenditure. TEE, total energy expenditure. TG, triglyceride. T2D, type 2 diabetes. VLCD, very low-carbohydrate diet. VLCK, very low-calorie-ketogenic. VLCKD, very low-carbohydrate ketogenic diet. VLDL, very low-density lipoprotein.

## 5. Cytokines That Burn Our Fire: Are They the Cornerstones of Obesity Treatment for the Foreseeable Future?

**Figure 1:**
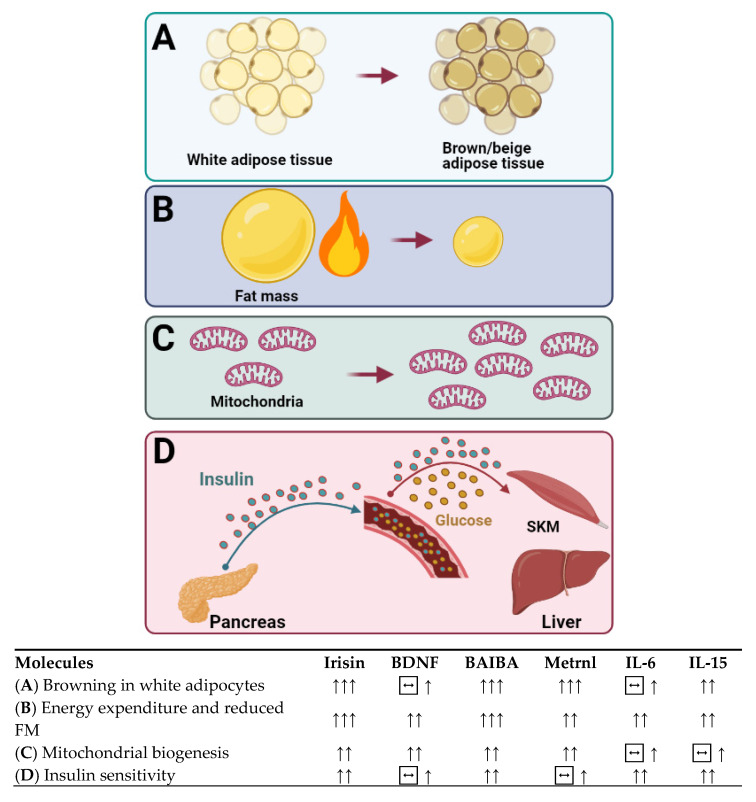
Potential role of exercise-induced myokines. ↑ values increase. 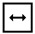 ↑ small change or no change. ↑↑ modest change. ↑↑↑ large change. FM fat mass. SKM skeletal muscle.

PA has been the cure for a healthy life and longevity since ~450 BC [216]. In the 1960s, with the technological advancements, the first scientific papers began publishing and provided mechanistic insight into how acute or chronic exercise could make a remarkable and paradigm shift in human physiology [217,218,219]. Although the existing literature attributes the health benefits of PA to reduced adiposity, increased cardiorespiratory fitness, reduced levels of circulating lipids, and the maintenance of muscle mass [220], the exact molecular mechanisms by which PA promotes human health is not fully elucidated. Moreover, over the last 20 years, considerable attention has been given to the interaction between skeletal muscle and the beneficial effects of exercise on health and compelling scientific evidence has proven that skeletal muscle is an endocrine organ in which various cytokines and peptides termed “myokines” are secreted. These molecules play a major role in the disease-preventative effects of regular PA [34]. Furthermore, it is well documented that proteins, peptides, enzymes, and metabolites released from other organs exert profound effects on several tissues including adipose tissue and skeletal muscle, and physiological functions, such as insulin sensitivity and glucose disposal. In this section, we will discuss some of these cytokines that have an important role in the therapeutic effects of exercise in the prevention of obesity. Potential role of exercise-induced myokines on browning of white adipocytes, energy expenditure, fat mass, mitochondrial biogenesis, and insulin sensitivity are presented schematically in Figure 1.

### 5.1. Brain-Derived Neurotrophic Factor

As a member of the neuro-trophin family, BDNF is a small, basic protein expressed in the brain [221] and, to a lesser extent, in skeletal muscle [222]. BDNF plays a primary role in the regulation of neuronal development, growth, and maintenance of neurons and modulates synaptic plasticity in the central nervous system (CNS) [223]. In addition to the essential role of BDNF in the CNS, it is involved in the control of BW, energy homeostasis, and EE in mice [224]. For example, obesity phenotypes, hyper-insulinaemia, and hyperglycemia were observed in mice with the BDNF gene deleted in excitatory neurons in the brain [225] and reduction in BDNF activity was shown to lead to abnormalities in eating behaviour [226], indicating a novel function of BDNF for energy homeostasis. In agreement with this finding, Lyons et al. reported that mice with global deletion of BDNF gene had, on average, 34% higher BW due to increased BF content as the primary cause and consuming 25% more food than their wild-type littermates [227]. Similarly, Yong et al. revealed that deleting the BDNF gene in the adult ventromedial hypothalamus resulted in significant hyperphagia and obesity in mice [228]. In support of this, Wang et al. showed that administration of BDNF in the hypothalamic paraventricular nucleus (PVN) plays an essential role in the regulation of energy metabolism, reduces BW due to a decrease in food intake, and an increase in EE [229], showing a novel role for BDNF in the PVN and the components of energy balance. Collectively, ample evidence suggests the key role of the BDNF gene in obesity. Furthermore, similar to exercise [230,231,232,233], central and peripheral exogenous BDNF treatments are known to decrease food intake, increase EE, reduce body weight, ameliorate hyper-insulinaemia, and hyperglycemia in mice [226,227], partly by inducing UCP1 gene expression reported to increase in BAT by 53.3% following administration of BDNF [229]. In addition, Cheng et al. reported that BDNF stimulated PGC-1α-dependent mitochondrial biogenesis, as indicated by increases in mitochondrial mass and upregulation of PGC-1α promoter activity and transcription and expression of PGC-1α [234]. These findings show that BDNF seems to be a therapeutic adjunct in the treatment of obesity. In humans, evidence-based research has revealed that the systemic level of BDNF is lower in obese people and T2D when compared to healthy individuals [235,236]. This case was also reported to impair glucose metabolism [235].

There has been growing interest in the effects of exercise on BDNF and in the role of BDNF in therapeutic effects of exercise since the elegant study of Neeper et al. reported for the first time that exercise increased BDNF gene expression in specific brain regions [237]. In the following years, it was reported that exercise can increase BDNF levels in rats [238] and humans [222]. A meta-analysis that reviewed 29 published papers showed that aerobic but not RT can increase resting BDNF concentration in peripheral blood in humans independent of exercise duration, intensity, and session time [239]. Another meta-analysis by Szuhany et al. showed that a single session of exercise can result in increased BNDF concentration in humans [240], suggesting acute exercise-induced upregulation of BDNF through which exercise is likely to improve cognitive function. Importantly, considering the lack of central function of BDNF leads to obesity [225], exercise intervention that results in increased BDNF should be encouraged to prevent obesity for all age groups. A study by Matthews et al. aimed to determine the signalling processes of BDNF following exercise intervention in humans, and reported that BDNF is an exercise-inducible protein that increases lipid oxidation in skeletal muscle by activating AMP-activated protein kinase (AMPK) [222], which is an important molecule in energy homeostasis [241]. However, since significant amounts of muscle-derived BDNF was not released into the circulation following exercise [222], it is tempting to speculate that there must be other sources responsible for the increased BDNF following exercise. For example, it has recently been shown that an exercise-induced increase in BDNF in the mouse brain is mediated by PGC-1α known to increase in response to exercise [242]. Moreover, platelets that are known to store BDNF and release it upon agonist stimulation are another alternative source of enhanced BDNF in the periphery following exercise, as shown in humans [243].

In summary, when BDNF was first discovered, its role in cognitive functions was emphasized in particular. In the following years, it was shown that BDNF is also an important regulator of metabolism. Although there has been some encouraging progress to unravel the mechanism through which BDNF affects energy metabolism, we are still far from a complete understanding of the role of BDNF in metabolism. Therefore, the essential function of BDNF in metabolism and exercise-induced changes in BDNF in the brain and skeletal muscle seem to be a fertile area of research for preventing and treating obesity. Further studies that reveal the relationship between BDNF and other molecules involved in metabolism and adipose tissue are also warranted.

### 5.2. β-aminoisobutyric Acid

β-Aminoisobutyric acid (BAIBA) is a nonprotein β-amino acid identified for the first time in human urine in 1951 [244], which is a recently discovered small myokine produced and secreted by skeletal muscle, as shown both in mice and human [245], and exerts either paracrine or endocrine effects to alter the physiological functions of target tissues [246]. BAIBA has two enantiomers in biological systems as R-BAIBA (D-BAIBA) and S-BAIBA (L-BAIBA) [247] and the total amount of BAIBA in human plasma was shown to consist of 98% R-BAIBA and only 2% S-BAIBA [248]. Studies that aimed to reveal the effects of BAIBA on metabolism showed that BAIBA is a myokine controlled by PGC-1α and acts in a myokine-specific manner [245], explaining lower plasma BAIBA in individuals with advanced age than young subjects [249].

Both animal and human studies suggest that BAIBA increases fat oxidation, reduces fat mass, improves glucose homeostasis, and prevents diet-induced obesity. In this regard, Begriche and colleagues showed that BAIBA significantly prevented diet-induced obesity, glucose intolerance, and hypertriglyceridemia in mice treated with BAIBA [250]. Another finding of this study was that BAIBA mediated its function through a leptin-dependent stimulation of mitochondrial fat oxidation [250]. Furthermore, chronic administration of BAIBA was reported to be an effective way to reduce fat mass [245], inducing browning of white fat tissue by increasing the expression of brown adipocyte-specific genes [245] and fat oxidation [251]. More recently, a pioneering study by Robert et al. documented that BAIBA markedly induced the gene expression of the mitochondrial UCP1 and mitochondrial biogenesis transcription coactivator PGC-1α [245] associated with BAIBA-induced fat loss. These effects of BAIBA were mediated via peroxisome proliferator-activated receptor alpha (PPARα), which plays a primary role in increasing fat oxidation and BAT metabolism [245]. Furthermore, BAIBA increased liver fatty acid oxidation and decreased hepatic lipogenesis by activating the transcription factor PPARα [252], improving insulin sensitivity and protecting against a high-fat diet-induced obesity [247,253,254]. This indicates the role of BAIBA in hepatic lipid metabolism and reduces risk of diabetes. More work is needed to reveal how BAIBA has a direct action on insulin signalling. Moreover, it has recently been reported that BAIBA can function as an osteocyte protective factor against mitochondrial degradation due to reactive oxygen species and reduce bone and muscle loss, resulting in hindlimb unloading [255]. Emerging evidence shows that BAIBA leads to increased hepatic fat oxidation and mRNA levels of the carnitine palmitoyltransferase 1 in hepatocytes, a rate-limiting β-oxidation enzyme [256], which resulted in reduced fat mass in human [251], showing a key role of BAIBA in reducing adipose tissue in humans as well.

Furthermore, BAIBA is generated by catabolism of the branched-chain amino acid valine that is mainly used in skeletal muscle [245], and catabolism of the branched-chain amino acids are elevated during exercise [257]. These findings are supported by research reporting that regular exercise increases circulating levels of BAIBA in previously sedentary and healthy subjects [245,248]. This increase is inversely associated with cardiometabolic risk factors in humans [245,247], suggesting that exercise-induced circulating BAIBA may play a role in the treatment of metabolic diseases. For example, Stautemas et al. investigated how an acute session of moderate-intensity exercise would affect the enantiomers of BAIBA [248]. They reported that R-BAIBA and S-BAIBA increased following 30 min of cycling, indicating BAIBA to be an acute exercise-induced molecule [248]. Similarly, Robert et al. showed a 20% increase in plasma BAIBA concentration in mice with access to the working wheel and a 17% chronic elevation following 20 weeks of aerobic exercise in sedentary and healthy subjects [245]. In addition, Short et al. reported that 16 weeks of aerobic exercise training resulted in a 29% greater increase in BAIBA levels in the normal-weight individuals compared to the individuals with obesity [258].

In summary, BAIBA as PGC-1α-mediated and exercise-induced myokine seems to be a mechanistic component of the well-known protective role of exercise against the development of metabolic diseases including obesity.

### 5.3. Interleukin-6

IL-6 is a cytokine that is not only secreted by immune cells during inflammatory states [259] but also released by adipose tissue and by the working skeletal muscle during exercise [260] in the absence of inflammation. The elevated number of M1 macrophages in WAT with obesity is accepted as the main source of IL-6 [261]. A chronic, low-level increase in basal levels of plasma IL-6 is associated with obesity [262], physical inactivity [263], insulin resistance [264], T2D [265], and cardiovascular diseases [266]. Furthermore, the IL-6 level was reported to elevate in individuals with obesity and positively associated with a waist-to-hip ratio and BMI [267], and decrease with weight loss [262]. On the other hand, muscle-derived IL-6, which is the first myokine found to be secreted into the bloodstream in response to muscle contractions [260], is an important player implicated in the regulation of lipid homeostasis and energy metabolism [268,269].

The concentration of circulating IL-6 can increase up to 100-fold during acute exercise and consistently declines in the recovery period [270,271,272] in the absence of muscle damage, depending on the intensity and duration of exercise, in particular. Evidence-based research shows that muscle cells are the main but not the sole source of the increase in IL-6 during exercise [273]. Despite the studies showing the internal jugular vein [272] and adipose tissue [274] that may contribute to the IL-6 response in the circulation following exercise, other sites are not yet fully determined. Moreover, several pieces of evidence show that there is a negative association between the amount of PA and resting plasma IL-6 levels [269], while physical inactivity and metabolic syndrome are associated with high basal plasma levels of IL-6. Moreover, endurance training reduces basal levels of IL-6 and the exercise-induced increase in plasma IL-6 and muscular IL-6 mRNA levels [269].

Until the beginning of this millennium, this significant increase in IL-6 response was first thought to involve muscle damage in the working muscles and that the macrophages were responsible for this increase [271]. In the following years, however, it was reported that there was a marked increase in intramuscular IL-6 mRNA expression and protein when intramuscular glycogen levels were low, indicating that IL-6 might be an energy sensor during exercise [34,269]. This notion was supported by numerous studies reporting that exercise-induced increase in plasma IL-6 and release from contracting skeletal muscle in humans attenuated during exercise following glucose ingestion [269]. IL-6 has also been linked to obesity and glucose metabolism. An elegant study by Wallenius et al. showed, for the first time, that IL-6-deficient mice developed mature-onset obesity and glucose intolerance [275]. Moreover, when the transgenic mice were treated with IL-6 for ~3 weeks, BW significantly decreased [275]. In addition, acute administration of rat L6 muscle cells in vitro with IL-6 increases basal glucose uptake, the translocation of the glucose transporter glucose transporter 4 (GLUT4), insulin-stimulated glucose uptake in muscle cells, lipolysis, and fatty acid oxidation [276]. Taken together, IL-6 is a therapeutic target for the treatment and prevention of obesity.

These effects of IL-6 arose via AMPK, as the results were not evident in cells infected with a recombinant expressing dominant-negative AMPK [276]. Furthermore, infusion of recombinant human IL-6 into healthy individuals was reported to increase lipolysis without changing catecholamines, glucagon, or insulin and no adverse effects were observed [277]. Several studies have also reported that IL-6 is a substance capable of increasing intramyocellular [276] or whole-body fatty acid oxidation [259] via AMPK [278]. Petersen et al. questioned if IL-6 would exert direct effects on both lipolysis and fatty acid oxidation [277]. To address this, the authors conducted cell culture experiments and reported that infusion of IL-6 activated lipolysis in patients with T2D and healthy individuals [277], suggesting IL-6 as a lipolytic factor. Similarly, Khan et al. showed that infusion of IL-6 into healthy humans at a physiological level markedly induced lipolysis in skeletal muscle, but there was no change in adipose tissue [279]. These findings show that IL-6 exerts its profound lipolytic effect in the muscle. Moreover, infusion of recombinant human IL-6 into healthy individuals during a hyper-insulinaemic clamp was shown to enhance whole-body insulin sensitivity [276].

In summary, IL-6 has beneficial effects on metabolic functions. Compelling evidence shows that IL-6 increases EE, lipolysis, fat oxidation, and endogenous glucose output, which are all associated with insulin action and substrate homeostasis. Furthermore, activation of AMPK by IL-6 plays an essential role in modulating some of these metabolic effects induced by IL-6. Considering that the increase in IL-6 level, especially observed in response to exercise, reduces adipose tissue, IL-6 may be a target peptide in the prevention of obesity. Taken together, it is clear that IL-6 is a cytokine that possesses great importance for metabolic health.

### 5.4. Interleukin-15

IL-15 is a highly expressed cytokine in muscle cells and, to a lesser extent, in multiple types of cells such as macrophages, fibroblasts, epithelial cells, keratinocytes, astrocytes, and bone marrow stromal cells [280,281]. The expression of IL-15 varies depending on the activity of the cell in which it is expressed. IL-15 expression is induced by nuclear factor kappa B (NF-kB) activators in macrophages [282], and by exercise intervention in muscle myotubes [283], making IL-15 an exercise-induced myokine. However, it is incompletely understood whether circulating IL-15 is released from skeletal muscle tissue in response to exercise or other physiological stimuli. In addition to IL-15, the IL-15 receptor-alpha (IL-15Rα) subunit is a primary binding partner of IL-15 and has complex biochemistry able to modulate IL-15 secretion and bioactivity. A study by Bergamaschi has documented that IL-15 was rapidly degraded immediately after synthesis, when the expression of IL-15Rα was blocked [284], showing the primary role of IL-15Rα in efficient IL-15 production. Moreover, IL-15 and IL-15 receptor subunit alpha (IL15RA) that encodes IL-15Rα were reported to be associated with increased adipocyte size and T2D [285].

Recently, IL-15 has attracted much attention from researchers due to its role in increasing EE and improving insulin sensitivity. These novel roles of IL-15 are associated with the endocrine roles of the myokines in metabolism [34]. Accumulating evidence has shown that overexpression of IL-15 is associated with brown fat function, reduced adiposity, and improved insulin sensitivity through weight loss and increased EE [286], suggesting IL-15 to function as a myokine able to mediate its effects on different tissues. Moreover, IL-15 is also known to enhance fat oxidation [287], glucose uptake [288], and myogenesis in skeletal muscle. In addition, circulating IL-15 has been reported to reduce lipid synthesis in preadipocytes in vitro and WAT in rats [289]. Furthermore, in humans, the plasma IL-15 level was shown to reduce BW [290] and be negatively associated with total fat mass, trunk fat mass, and percent fat mass in individuals with or without obesity [290], indicating a novel role for IL-15 in regulating fat mass and adiposity. In support of this finding, Alvarez et al. reported that acute injection of recombinant IL-15 into rodent genetic obesity models inhibited fat deposition in both wild-type and leptin-deficient obese mice [291]. Conversely, mice with the IL15 gene deleted in cultured adipocytes showed higher amounts of BF than control mice [292], whereas transgenic mice with elevated circulating levels of IL-15 had lower levels of BF and were resistant to diet-induced obesity [286]. Moreover, it was reported that people with T2D had lower circulating IL-15 levels compared with body-weight-matched healthy controls [293,294]. Some research, however, has documented that IL-15 remained unchanged by insulin resistance [290] and people with T2D exhibited higher serum IL-15 than healthy controls [294]. It is, however, necessary to denote here that PA, adiposity, and age that may alter circulating IL-15 levels are not controlled in these mentioned studies, necessitating further studies able to consider confounding factors.

In recent years, there has been increasing interest in the role of IL-15 in the beneficial effects of exercise. Numerous studies have been conducted to address this. Yet, there is no clear consensus on whether acute or chronic exercise intervention can alter mRNA, protein, and circulating level of IL-15. For example, circulating IL-15 levels showed a transient increase following acute RE [295], but did not change with training [295]. Nielsen et al. investigated the effects of an acute heavy RE session on a leg press machine and then on a knee extensor machine [296]. They reported upregulation of IL-15 mRNA at 24 h of recovery following the exercise session and the IL-15 mRNA levels returned to pre-exercise levels 48 h after the end of the exercise, whereas muscle IL-15 protein content and plasma IL-15 concentrations were similar between pre-exercise and 6, 24, and 48 h post-exercise [296]. Notably, the authors also showed, for the first time, that IL-15 mRNA levels were higher in the triceps compared to the soleus muscle, showing that RT-induced change in IL-15 mRNA levels may be observed specifically in type 2 fibres.

Some research has focused on the effects of acute and chronic endurance training on IL-15 levels. For example, 30 min, 60 min, or 2 h of moderate intensity running or cycling exercise resulted in a relatively transient increase in serum IL-15 levels measured 10, 30, and 120 min after the end of the exercise in lean individuals and those with obesity [283,297]. However, Rinnov et al. and Ostrowski et al. found no change in circulating IL-15 levels following 2.5–3 h of aerobic exercise at ~60–75% of VO2_max_ [298,299], but there was a 40% increase in basal skeletal muscle’s IL-15 protein content following 12 weeks of regular endurance training (5 days/week) [298] with no change in either muscle IL-15 mRNA or plasma IL-15 levels. In contrast, Pérez-López et al. reported reduced IL-15 level in individuals with or without obesity, who were engaged in regular PA (3 days/week) for one year [294]. While it is difficult to discern the reasons for this absence of increased IL-15 following acute exercise, the timing of the blood sampling may be important in detecting the exercise response, especially when considering that the half-life of free IL-15 is about 30–60 min [280,300]. Therefore, studies that measured IL-15 not immediately after exercise but >60 min after exercise are likely to miss the peak of IL-15 increase.

In summary, the molecular mechanism of IL-15 action is not fully uncovered in the regulation of energy metabolism. However, current evidence shows IL-15 to play an essential role in adiposity and EE, making IL-15 one of the novel targets for pharmacologic control of obesity. In addition, the effect of exercise on IL-15 is largely unresolved and awaits determination whether it varies in different types of contractions/exercise.

### 5.5. Irisin

Adipose tissue and skeletal muscle are endocrine organs capable of secreting many bioactive molecules [231]. Molecules secreted from skeletal muscle are called myokine and molecules secreted from adipose tissue are called adipokine [301]. Accordingly, hormones secreted from both are called adipo-myokine [302]. As an adipo-myokine, irisin secreted from adipose tissue is only 5% of the level of irisin secreted from the skeletal muscle [141]. Irisin is also secreted from the heart muscle, liver, brain, pancreas, and kidney [303,304]. Irisin is an exercise-induced myokine derived from fibronectin type III domain-containing 5 (FNDC5) [305,306,307]. As a PGC-1α-dependent myokine [307], irisin drives brown-fat-like thermogenesis in WAT in rodents [307] and humans [308] and promotes mitochondrial biogenesis [309], and decreases oxidative stress [310]. In recent years, it has become apparent that irisin as a cleaved fragment is also secreted into the circulation following proteolytic cleavage from its cellular form in humans and can readily be quantified by an enzyme-linked immunosorbent assay [311]. Evidence-based research has revealed that irisin can reverse diet-induced obesity and diabetes by stimulating thermogenesis in rodents [307] and humans [308] by increasing brown adipocyte-like cell abundance and increasing the expression of brown adipocyte-specific genes [307] within WAT that, in turn, increases EE [312].

Adipose tissue is the second main source of irisin. Importantly, irisin was shown to release from mature adipocytes of WAT in rats, which is mainly from those in SAT and a lower amount from those in VAT [313]. WAT-derived FNDC5/irisin represents ~28% of total circulating levels of the protein, with the remaining 72% likely derived from skeletal muscle [307,313,314], indicating that adipose tissue is not an essential source of irisin. The expression of FNDC5 in adipose tissue is about 100–200 times lower than in skeletal muscle in humans [304,315]. It is also well documented that irisin exerts its profound effect on adipose tissue depending on the species (rodents, humans), type of adipocytes (premature or mature adipocytes), and location/type of the adipose tissue [316].

The effects of irisin on the browning of WAT in humans are incompletely understood. Irisin decreases browning-related genes in human preadipocytes without stimulating browning in human preadipocytes from SAT, whereas it stimulates browning, indicated by an increase in UCP1 and various signalling pathways in mature human adipocytes [308,317]. Moreover, the potential correlations between circulating irisin levels and obesity have been investigated extensively. Studies showed a positive association between circulating irisin and BMI, BW, waist circumference, and waist-to-hip ratio [304,318], even though some research reported a negative correlation between irisin and BMI [319]. Moreover, Zhang et al. [320] demonstrated that recombinant irisin resulted in decreased BW and improved glucose homeostasis. They also showed that irisin stimulated UCP-1 expression and the expression of betatrophin, which is a hormone that promotes pancreatic β-cell proliferation and improves glucose tolerance [320]. In addition, mice adipocytes treated with intravenous injection of FNDC5/irisin exhibited multilocular lipid droplets, a higher density of mitochondria, and increased EE, showing induction of the brown adipocyte-like phenotype in WAT [307]. Irisin has also been shown to induce lipid metabolism and downregulate lipid synthesis in mice [321] and is positively associated with biceps circumference, fat-free mass, and BMI in humans [304]. Park et al. [322] also assessed if circulating irisin was associated with macronutrients, energy intake, and dietary scores and reported that irisin was not associated with any of the studied dietary factors including the Alternate Healthy Eating Index and Alternate Mediterranean Diet Score [322]. Crujeiras et al. [323] reported that, in addition to the well-studied hormones leptin and adiponectin, irisin plasma levels were also associated with insulin resistance in weight regainers versus non-regainers, indicating irisin to be a potential prognostic marker of T2D. Collectively, irisin seems to have a key role in the improvement of adipocyte metabolism and can serve as a potential therapeutic target in the future.

The molecular mechanism underpinning exercise-induced irisin concentration is that exercise increases the expression of PGC-1α, which leads to the expression of FNDC5, the precursor of irisin, in the brain and skeletal muscle [307]. Irisin is cleaved from FNCD5 at the level of the cell membrane by the unknown enzyme(s) and then binds to yet undefined receptor(s) of white adipocytes and other cells [314]. Irisin stimulates the expression of mitochondrial UCP1 and browning of WAT [324], which is known to induce thermogenesis and, thus, EE in the skeletal muscle and BAT [312,316]. Many studies examined the effects of different types of exercise on circulating irisin in humans with inconsistent results. It has been reported that chronic exercise decreases [325,326,327,328,329], increases [330,331], and does not change the resting irisin concentration [141,332]. Eight weeks of RT significantly increased circulating irisin, while there was no change in irisin following aerobic training [333]. Similarly, Tsuchiya et al. [334] showed that acute RT increased circulating irisin more than endurance or combined resistance/endurance training in healthy individuals, showing that RT could provide more stimulus to induce irisin in humans than any other type of exercise. Additionally, a higher increase in irisin concentration was reported following high-intensity acute exercise compared to low-intensity exercise [335]. Two meta-analyses conducted on the effects of exercise on irisin concentration have shown that acute exercise [306] and chronic RT can increase irisin level based on the exercise protocol applied [305], while endurance training decreases irisin concentration negligibly [306], showing that irisin responses to exercise are acute and physiological adaptations following chronic exercise are not sufficient to keep the resting irisin level high. Speculatively, this decrease in irisin level following endurance training may be due to increased sensitivity of the unknown irisin receptor in response to exercise [327].

In summary, irisin is a potential mediator of the health-promoting effects of exercise. Due to its effects on metabolism and adipose tissue, irisin is considered a promising therapeutic target for treating obesity and T2D.

### 5.6. Meteorin-Like

Meteorin-like (Metrnl) is a circulating factor, which is induced in muscle in response to exercise and in adipose tissue upon cold exposure, and is involved with mitochondrial biogenesis in white adipocytes [336]. The highest expression level of Metrnl is in WAT of both rodents and humans, with expression in various tissues including omental adipose tissue, perivascular adipose tissue, interscapular adipose tissue, liver, spleen, muscle, heart, thymus, forebrain, midbrain, and hindbrain [337]. Metrnl is known to prevent insulin resistance induced by a high-fat diet or leptin deletion [338], regulate immune-adipose interactions, contribute to browning of the subcutaneous WAT [336], improve adipose tissue function [337], promote neurite outgrowth [339], and increase systemic EE [336]. These findings indicate Metrnl plays a potential role in preventing metabolic diseases and improving metabolism. Recently, the Spiegelman group has shown that Metrnl increased the production of IL-4, IL-13, and catecholamines in the adipose tissue in vivo, showing Metrnl-induced phenotypic switch in adipose tissue macrophages and production of pro-thermogenic catecholamines [336]. Moreover, the same research group has documented that increased Metrnl plasma concentration resulted in increased EE, and improved glucose tolerance and anti-inflammatory cytokines [336]. In addition, the plasma Metrnl level has been reported to be lower in people with T2D [340] and osteoarthritis [341] when compared to healthy subjects. On the other hand, some research reported a higher level of Metrnl in individuals with obesity [342], and a positive association of Metrnl with BMI, waist circumference, total cholesterol, triglyceride, and low-density lipoprotein cholesterol in individuals with T2D [342,343]. These discrepancies available in the literature remain paradoxical and await clarification.

Supporting evidence has shown that Metrnl is regulated by acute and chronic exercise intervention. For example, Rao et al. reported a significant increase in Metrnl mRNA expression in the triceps muscle of mice and a two-fold increase in circulating Metrnl concentration following a single bout of downhill treadmill-running exercise [336]. The same study also showed that an acute session of CT consisting of RE followed by endurance exercise increased Metrnl mRNA expression in human skeletal muscle [336]. Concerning the training effects on Metrnl, Bae reported that chronic treadmill running (5 sessions/week for 8 weeks) significantly increased muscle, plasma, and adipose tissue Metrnl in high-fat diet-induced obese mice and reduced high-fat, diet-induced BW gain in mice without affecting caloric intake [344]. Similarly, Amano et al. reported an increased plasma Metrnl in mice following four weeks of chronic RT administered with electrical stimulation and this increase was associated with an increase in the expression of PGC-1α and mitochondrial biogenesis in BAT [345]. Furthermore, Eaton et al. reported that a single bout of high-intensity interval exercise and 20 days of HIIT significantly increased Metrnl mRNA expression in human skeletal muscle [346], indicating Metrnl mRNA expression is responsive to the acute and chronic high-intensity exercise intervention. However, it remains uncertain whether acute or chronic exercise intervention would affect Metrnl at the protein level in muscle or plasma.

In summary, Metrnl is an exercise-induced protein that promotes the expression of genes associated with the browning of WAT. This transformation of characteristic white adipocytes into brown/beige fat is of great therapeutic potential in developing new therapies for obesity and T2D. Therefore, exercise interventions that increase circulating concentration and mRNA expression of Metrnl would be novel therapeutic strategies to overcome chronic diseases, particularly obesity. Furthermore, clarifying the roles and possible clinical applications of Metrnl in more detail may alleviate obesity and offer protection against metabolic disorders due to the essential role of Metrnl in neurotrophic activity and metabolism.

## 6. Summary and Future Perspectives

In the present study, we reviewed the therapeutic roles of various diet, exercise interventions, and some cytokines that play a significant role in obesity prevention and improving adipose tissue metabolism. Obesity has been an epidemic disease defined as an excess of BF that results from a state of positive energy balance in which energy intake exceeds the expenditure. The cause of obesity is well known to be multifactorial including genetics, nutrition, and lack of PA. Thus, this complexity must be taken into account when developing preventive interventions. The most important ways to reverse the increased prevalence of obesity are dependent on the interventions that provide a sustained negative energy balance over time. From this point of view, regular exercise seems like an elixir capable of leading to a negative energy balance that reduces both subcutaneous and VAT mass. Furthermore, a combined hypocaloric diet and PA intervention is likely to be more effective in reducing BW and improving adipose tissue metabolism. Notably, even if there is no weight loss despite exercise training, regular exercise is a cornerstone providing numerous benefits, such as maintenance of muscle mass and RMR, inducing anti-inflammatory markers, improving skeletal health, and more. Likewise, cytokines and myokines secreted from various organs and tissues establish a muscle-to-organ/tissue cross-talk communication that promotes health-related outcomes. These cytokines and myokines known to be secreted in response to muscular contractions are promising molecules for the prevention of obesity particularly due to their ability of browning white fat tissue by increasing the expression of specific genes within WAT. However, the role of most myokines is not fully elucidated. Therefore, more work is needed to provide a better understanding of the physiological role of myokines in humans.

## Data Availability

Not applicable.
